# A Comparative Study of Two Synthesis Methods for Poly(Acrylic Acid-*Co*-Acrylamide) Incorporating a Hyperbranched Star-Shaped Monomer

**DOI:** 10.3390/polym17070964

**Published:** 2025-04-01

**Authors:** Ramses S. Meleán Brito, Agustín Iborra, Juan M. Padró, Cristian Villa-Pérez, Miriam C. Strumia, Facundo Mattea, Juan M. Giussi, Juan M. Milanesio

**Affiliations:** 1 Departamento de Química Orgánica, Facultad de Ciencias Químicas, Universidad Nacional de Córdoba, Av. Haya de la Torre y Av. Medina Allende, Córdoba X5000HUA, Argentina; ramses.melean@unc.edu.ar (R.S.M.B.); mstrumia@unc.edu.ar (M.C.S.); fmattea@unc.edu.ar (F.M.); 2 Instituto de Investigación y Desarrollo en Ingeniería de Procesos y Química Aplicada (IPQA—UNC—CONICET), CONICET, Av. Vélez Sarsfield 1611, Córdoba X5016GCA, Argentina; 3 YPF TECNOLOGÍA S.A., Av. Del Petróleo s/n (entre 129 y 143), Berisso B1923, Argentina; agustin.iborra@ypftecnologia.com (A.I.); juan.padro@ypftecnologia.com (J.M.P.); cristian.villa.p@ypftecnologia.com (C.V.-P.); juan.m.giussi@ypftecnologia.com (J.M.G.); 4 Departamento de Química, Facultad de Ciencias Exactas, Universidad Nacional de La Plata (UNLP), 47 and 115, La Plata B1900AJL, Argentina; 5 Departamento de Química Industrial y Aplicada, Facultad de Ciencias Exactas Físicas y Naturales, Universidad Nacional de Córdoba, Av. Vélez Sarsfield 1611, Córdoba X5016GCA, Argentina

**Keywords:** free radical polymerization, associative polymers, supercritical fluids, macromonomer

## Abstract

The synthesis of poly(acrylic acid-*co*-acrylamide) was investigated to enhance its rheological properties. Syntheses were conducted in both aqueous and supercritical fluid media, with and without the incorporation of a novel star-shaped macromonomer. The macromonomer, synthesized from a Boltorn H30 core with PEGMA^500^ arms and modified to contain a single vinyl group, was copolymerized with acrylic acid and acrylamide. Comprehensive polymer characterization was performed using FTIR, NMR, and SEC-MALS-dRI techniques. Rheological assessments revealed that copolymers containing the star-shaped monomer exhibited significantly higher viscosities than those lacking the hyperbranched component, a result attributed to the inter- and intrachain interactions facilitated by the PEGMA^500^ arms. Additionally, purification studies demonstrated that dialysis was necessary to remove short-chain polymers, particularly for samples synthesized in supercritical media, to achieve optimal rheological performance. Polymers synthesized in a supercritical CO_2_–ethyl acetate mixture exhibited higher viscosities compared to their water-synthesized counterparts. The integration of the novel star-shaped macromonomer into HPAM-like polymers offers substantial potential for enhanced oil recovery applications.

## 1. Introduction

Partially hydrolyzed polyacrylamide or poly(acrylic acid-*co*-acrylamide) (HPAM) is widely used in enhanced oil recovery (EOR) due to its cost-effectiveness, high viscosity-enhancing properties, and resistance to mechanical, oxidative, and thermal degradation [1]. EOR processes often rely on polymers to increase the viscosity of injected water, improving mobility ratios, reducing water consumption, and suppressing viscous fingering [2]. While both natural and synthetic polymers have been explored for EOR, HPAM’s effectiveness is limited under the harsh conditions of many reservoirs [3]. High salinity and elevated temperatures [4,5] can significantly reduce HPAM viscosity, negatively impacting its performance and long-term efficacy [6]. As a result, a key area of EOR research focuses on developing novel HPAM-based copolymers that maintain or enhance viscosity under these challenging conditions [7].

One approach to improving HPAM performance involves chemical modification [8]. Studies have shown that viscosity can be enhanced through techniques such as grafting with chitosan and hydrophobic molecules [9], incorporating hydrophobic monomers [10,11], and synthesizing hyperbranched polymers [12]. Dendrimer modifications have also shown promise, particularly for heavy oil applications [13]. Hyperbranched polymers and molecules, characterized by their intricate molecular architectures and abundant terminal functionalities, offer significant versatility in material design [14,15]. The detailed reported synthesis pathways highlight the ability to precisely tailor key properties, such as viscosity, thermal stability, and chemical resistance, by controlling branching density and functional group incorporation [15,16,17]. In this regard, hyperbranched Boltorn H30 serves as a versatile building block [18,19,20]. Its numerous hydroxyl groups facilitate chemical modification and associative interactions, making it an ideal candidate for macromonomer synthesis. The incorporation of poly(ethylene glycol) methyl ether methacrylate (PEGMA), known for its stabilizing effects [21], onto Boltorn H30-derived macromonomers can be used to modify HPAM copolymers with improved rheological properties in high-salinity and high-temperature environments [22].

Supercritical CO_2_ (scCO_2_) presents potential advantages as a polymerization medium [23,24,25,26,27]. Its non-toxic and non-flammable nature, along with its readily achievable critical conditions and unique combination of gas-like diffusivity and liquid-like density, can enhance reaction kinetics, minimize solvent-related issues, and simplify polymer separation and recycling. Conventional HPAM is typically synthesized in solution, inverse emulsion, dispersion, or precipitation polymerization [8]. However, the extremely low solubility of acrylamide in scCO_2_ [28] has hindered the direct use of scCO_2_ for HPAM synthesis. Nevertheless, incorporating a co-solvent in precipitation polymerization may provide a viable pathway.

This study explores for the first time the polymerization of acrylic acid, acrylamide, and a custom designed hyperbranched macromonomer by iodine transfer radical polymerization in supercritical fluids. Additionally, the results are compared to the same polymer synthesized in aqueous media by following the procedure previously reported [22]. The research examines how these synthetic strategies influence the molecular characteristics and macroscopic properties of the resulting copolymers.

## 2. Materials and Methods

### 2.1. Materials

The following materials were used without any further purification or pretreatment: acrylic acid (AAc) with a purity of 98% (79-10-7 CAS number), acrylamide (AAm) with a purity of 99% (2094-98-6 CAS number), hyperbranched *bis*-MPA polyester-32-hydroxyl, generation 3 or Boltorn H30 “C_155_H_226_O_93_” (H30, 97.0%) (326794-48-3 CAS number), poly(ethylene glycol) methyl ether methacrylate (PEGMA^500^, average *M_n_* 500 g mol^−1^) (26915-72-0 CAS number), *α*-bromoisobutyryl bromide (98%) (20769-85-1 CAS number), triethylamine (TEA, 99.5%) (121-44-8 CAS number), copper (I) bromide (CuBr, 98%) (7787-70-4 CAS number), *N*,*N*,*N*′,*N*″,*N*″-pentamethyldiethylenetriamine (PMDETA, 99%) (3030-47-5 CAS number), methacrylic anhydride (94%) (760-93-0 CAS number), iodine (99.8%) (7553-56-2 CAS number), deuterated water (D_2_O, 99.9%) (7789-20-0 CAS number), 1,1′-azobis(cyclohexanecarbonitrile) (ABCN, 98%) (2094-98-6 CAS number), potassium persulfate (KPS, 99%) (7727-21-1 CAS number), and *N*′-tetramethyl ethylenediamine (TEMED, 99%) (110-18-9 CAS number) were purchased from Sigma Aldrich (St. Louis, MO, USA). Sodium hydroxide (NaOH, 99%) (1310-73-2 CAS number) and ethyl acetate (EtAc) (141-78-6 CAS number) 99% pure were provided from Anedra (Buenos Aires, Argentina). Tetrahydrofuran HPLC grade (THF) (109-99-9 CAS number), dry dichloromethane (DCM) (MAX 0.005%water) (75-09-2 CAS number), absolute methanol (MeOH) (67-56-1 CAS number), and absolute ethanol (EtOH) (64-17-50 CAS number) were acquired from Cicarelli (Sante Fe, Argentina), and carbon dioxide (124-38-9 CAS number) with a purity of 99.995% (GC) was purchased from Linde (Buenos Aires, Argentina) and used as received.

All solutions were prepared with ultra-pure water (18 MΩ cm^−1^) from a Millipore Milli-Q^®^ system (Darmstadt, Germany).

### 2.2. Synthesis and Purification of Macroinitiator for ATRP and the Macromonomer Boltorn H30—PEGMA^500^—V (MM)

An ATRP macroinitiator was synthesized following the procedure reported previously [22] and briefly described here. Boltorn H30 was modified with α-bromoisobutyryl bromide using molar ratios of 4.5:1 of hydroxyl groups to α-bromoisobutyryl bromide, resulting in approximately 25% (8) of the hydroxyl groups being modified. For that purpose, Boltorn H30 (0.28 mmol, 1.0 g) was dissolved in ultra-dry THF and treated with TEA. The reaction mixture was cooled to 0 °C and α-bromoisobutyryl bromide (2.22 mmol, 0.255 g) was added dropwise under nitrogen (N_2_) atmosphere. After stirring at 0 °C for 2 h and at room temperature for 24 h, the precipitated salt was removed by filtration. The product was purified by precipitation in MeOH and dried under vacuum to yield 1.0 g of brominated Boltorn (Boltorn H30-Br). The Boltorn H30-Br macroinitiator was then grafted with 5.5 g of PEGMA^500^ via ATRP to obtain the macromonomer Boltorn H30-PEGMA^500^. The reaction used CuBr and PMDETA as a copper complexing agent in methanol under an N_2_ atmosphere [22] The PEGylation step conferred water solubility and enhanced salinity resistance to the macromonomer [21]. The product was purified by dialysis (14 kDa membrane cut-off) and lyophilization to yield 4.5 g of Boltorn H30-PEGMA^500^.

In designing the macromonomer, only eight of the 32 hydroxyl groups in Boltorn H30 were converted into ATRP-initiating groups. Due to the hyperbranched structure, only peripheral hydroxyls are readily accessible, and previous experiments [22] indicated that exceeding eight modifications compromises the yield and could increase the final application costs.

Boltorn H30-PEGMA^500^ was modified subsequently with 0.026 g of methacrylic anhydride to introduce a vinyl group by esterification of the remaining hydroxyl groups on Boltorn H30 [22,29]. The reaction used anhydrous DCM as the solvent and TEA as a catalyst. Methacrylic anhydride was added dropwise to a solution of Boltorn H30-PEGMA^500^ in DCM under an N_2_ atmosphere with stirring at 0 °C for 2 h. The reaction proceeded for 24 h at 30 °C and the remaining DCM was evaporated. The final product was purified by dialysis and lyophilization to yield 3.9 g of Boltorn H30–PEGMA^500^–V (MM). The methods for obtaining the macroinitiator and the macromonomer were extensively described elsewhere, including molecular schemes of the involved reactions [22]. The chemical structures of Boltorn H30 and the macromonomer Boltorn H30–PEGMA^500^–V are provided in Figures S1 and S2 of the Supporting Information.

### 2.3. Synthesis and Purification of Acrylamide, Acrylic Acid, and Boltorn H30—PEGMA^500^—V Terpolymer and Poly(Acrylamide-Co-Acrylic Acid) Copolymer

#### 2.3.1. Via Reverse Iodine Transfer Polymerization in Aqueous Medium (HPAM-H_2_O and HPAM-MM-H_2_O)

HPAM-MM-H_2_O and HPAM-H_2_O copolymers were synthesized by reverse iodine transfer polymerization (RITP) reaction in aqueous solution [30]. A monomeric mixture of AAm and AAc (3:1) was used, along with 0.85 wt% of the vinyl macromonomer MM. KPS and TEMED were used as thermal initiators, and the pH of the water solution was adjusted to 8 using NaOH. An ethanolic solution of iodine was introduced as a transfer controlling agent. The molar ratio of iodine to initiator was 1:5, indicating that one mole of iodine was added for every five moles of initiator. The reaction was conducted for 24 h at 65 °C under stirring. The same procedure and molar ratio were followed to obtain HPAM-H2O without the MM [22]. The chemical structures of the synthesized copolymers HPAM-H_2_O and HPAM-MM-H_2_O are shown in Figures S3 and S4 of the Supporting Information.

#### 2.3.2. Via Reverse Iodine Transfer Polymerization in Supercritical Carbon Dioxide + Ethyl Acetate Solvent Mixture (HPAM-CO_2_ and HPAM-MM-CO_2_)

HPAM-MM-CO_2_ and HPAM-CO_2_ copolymers were synthesized by RITP in pressurized CO_2_ + ethyl acetate solvent mixture (The chemical structures of the synthesized copolymers HPAM-CO_2_ and HPAM-MM-CO_2_ are shown in Figures S3 and S4 of the Supporting Information). A custom-built high-pressure stainless-steel reactor was used for these reactions. The reactor has a maximum pressure of 400 bar and a volume of approximately 150 mL. It is equipped with a magnetic stirrer and a circulating glycerin bath. The procedure followed was based on a previous work by Bouilhac et al. [31].

The reaction procedure starts with the addition of the monomeric mixture in an AAm:AAc known molar ratio of 3:1 for both copolymers and 0.85 wt% of the vinyl macromonomer MM, in the AAm:AAc:MM copolymer, followed by the addition of the thermal initiator ABCN, an ethanolic solution of iodine as a transfer control agent, and ethyl acetate as the cosolvent. The molar ratio of iodine to initiator was 1:5, indicating that one mole of iodine was added for every five moles of initiator. The amount of ethyl acetate added was sufficient to dissolve the acrylamide. At most, this amount was 25 mL. The reactor was then sealed and purged with CO_2_ at low pressure (2 bar) for 15 min at 45 °C. After tightening the top cap, CO_2_ was added to the reactor using a high-pressure syringe pump. Then, the reaction temperature was increased to 90 °C, maintaining a constant pressure of 320 bar inside the reactor.

**Figure 1:**
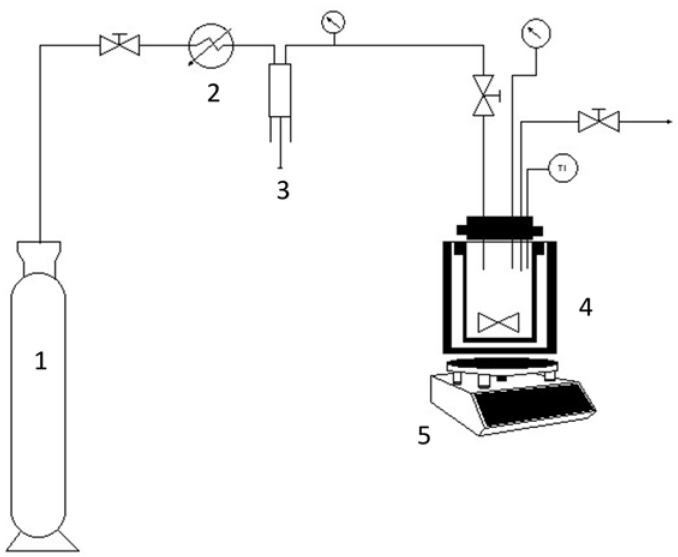
Schematics of the reaction setup. 1: CO_2_ bottle, 2: cooling system, 3: syringe pump, 4: high pressure reactor, and 5: magnetic stirrer.

The reaction proceeded for 12 h, with an initial pressure increase of 30–40 bar after the first hour of reaction. Then, the reactor was cooled, depressurized, and the product was collected. The yield was determined by considering the ^1^H NMR vinylic signals of the unreacted monomers. Figure 1 shows the experimental setup. Table 1 shows the molar ratios used in the reactions.

### 2.4. Purification of the Synthesized Copolymers

The synthesized HPAM-MM-H_2_O, HPAM-MM-CO_2_, HPAM-H_2_O and HPAM-CO_2_ copolymers were purified in two steps. First, the copolymers were purified by solvent precipitation. For that purpose, the samples were dissolved in ultra-pure water and then added dropwise to ethanol. The precipitated copolymers were washed with acetone and dried at 60 °C to remove the residual solvent. In the second step of purification, the previously precipitated copolymers were dialyzed (14 kDa membrane cut-off) and lyophilized at −86 °C and 0.041 mbar. A comparison of the results obtained through these two sequential purification steps was conducted, as notable differences were observed.

### 2.5. Characterization Techniques

Several analytical techniques were used to confirm the incorporation of MM into the polymer backbone. Proton nuclear magnetic resonance (^1^H NMR) spectroscopy was used to quantify the MM content in the copolymers. Size-exclusion chromatography coupled with multi-angle light scattering and differential refractive index (SEC-MALS-dRI) as well as dynamic light scattering (DLS) were used to determine the molar mass distribution (*MWD*) of the polymer and size after purification. The apparent viscosities of the polymer solutions in ultra-pure water were also measured at 2500 mg L^−1^.

#### 2.5.1. Fourier Transform Infrared Spectroscopy (FTIR)

FTIR spectra of the polymers were collected using a Nicolet iN10 spectrophotometer (Thermo Fisher Scientific, Waltham, MA, USA). The spectra were recorded in the range of 600–4000 cm^−1^, and each sample was scanned an average of 64 times. The samples were analyzed without any previous preparation on a gold plate using a Mercury–Cadmium–Telluride detector (Thermo Fisher Scientific, Waltham, MA, USA).

#### 2.5.2. Proton and Carbon Nuclear Magnetic Resonance Spectroscopy (^1^H and ^13^C NMR)

^1^H and ^13^C NMR spectra were obtained with a Bruker Ascend 500WB spectrometer (Bruker Corporation, Billerica, MA, USA) equipped with a 10 mm BBO probe and an Avance III HD console (Bruker Corporation, Billerica, MA, USA), operating at 500 MHz for ^1^H or 125 MHz for ^13^C, using D_2_O as the solvent for the copolymers. For the ^1^H spectra, 1024 transients were accumulated with a relaxation delay of 10 s and a 30° flip angle. The Inverse Gated ^1^H decoupling technique (zgig pulse sequence) was used to obtain quantitative ^13^C NMR spectra; 16,384 scans were acquired with a relaxation delay of 10 s. Chemical shifts are documented in parts per million (δ, ppm) and internally referenced to residual solvent signals. ^1^H NMR measurements were performed on the samples after synthesis without purification to determine the percentage of unreacted monomers, and after the purification process to quantify the MM content in the copolymers. All the spectra were measured at 25 °C.

#### 2.5.3. Differential Scanning Calorimetry (DSC)

DSC was performed using a TA instrument (DSC 250, New Castle, DE, USA) with aluminum crimped pans at a heating rate of 5 °C min^−1^, under nitrogen atmosphere with a flowrate of 50 mL min^−1^ and a temperature range of 20 to 400 °C.

#### 2.5.4. Scanning Electron Microscopy (SEM)

The morphology of the lyophilized polymers was examined using a FEI Quanta 200 SEM microscope (FEI Company, Hillsboro, OR, USA). This instrument utilizes a tungsten thermionic emitter (W) to generate the electron beam, which can operate within a voltage range of 200 V to 30 kV. The analysis was conducted under a vacuum of 10^−4^ Pa, with secondary electron detection performed using an SDD/Bruker 30 mm^2^ detector (Bruker Corporation, Billerica, MA, USA) to capture the polymer morphology. Prior to the analysis, the samples were coated with a 3 nm layer of platinum applied over an adhesive film.

#### 2.5.5. Size Exclusion Chromatography with Coupled Multi-Angle Light Scattering and Differential Refractometer Index Detectors (SEC-MALS-dRI)

SEC-MALS-dRI was performed to determine the weight-average molar mass (*Mw*), the molar mass distributions, and the radius of gyration (*R_g_*) of the polymers after purification for precipitation and dialysis of the polymer samples, to determine the effect of the purification process on the molar mass distributions. The SEC-MALS-dRI consisted of a separation module with a vacuum degasser, a quaternary pump, an autosampler, and a thermostatted column device SCL-40 System Controller (U-HPLC Nexera, Shimadzu, Kyoto, Japan) connected to a detector train consisting of a DAWN 8 MALS detector (Wyatt Technology Corp., Santa Barbara, CA, USA) simultaneously measuring the scattered light at 8 angles with nominal values ranging from 28° to 147°; followed by a differential refractive index detector (dRI) Optilab (Wyatt Technology Corp., Santa Barbara, CA, USA). For this, the 90° detector was previously calibrated with toluene, a highly scattering solvent with a known Rayleigh ratio, according to the manufacturer’s instructions, obtaining a calibration constant equal to 5.8293 × 10^−5^ (V m^−1^). The dRI detector was calibrated with NaCl standards over a linear concentration range of 1–5 mg mL^−1^, operated at 20 °C, and yielded a calibration constant of 3.4345 × 10^−5^ V refractive-index unit (RIU)^−1^. Bovine serum albumin (BSA monomer), 2 mg mL^−1^ in a 0.9% NaCl solution containing sodium azide, M = 66.4 kDa, R_h_ = 3.5 nm, acquired from Thermo Scientific^®^ (Waltham, MA, USA), was used for normalization coefficients for light scattering, performing peak alignment (inter-detector delay volumes), and band broadening correction parameter procedures. The separation was performed using a set of three analytical SEC columns with hydroxylated methacrylate phase: TSKgel G4000PW_xl_ (300 × 8 mm, 10 μm), TSKgel G5000PW_xl_ (300 × 8 mm, 10 μm), and TSKgel G6000PW_xl_ (300 × 8 mm, 13 μm) with calibration range: 2000–3.0 × 10^5^; 4000–8.0 × 10^5^, and 4.0 × 10^4^–8.0 × 10^6^ Da, respectively (polyethylene glycols and oxides) (TOSOH Biosciences, Tokyo, Japan), and a flow-rate of 1.00 mL min^−1^ was used in all analyses. The column temperature was set at 20 °C. Detector temperatures were maintained at 20 °C and the injection volume was 50 μL.

Aqueous-phase SEC analysis samples were prepared at a concentration of 0.5 mg mL^−1^ and at least 48 h prior to the SEC analysis dissolved and analyzed in an aqueous phosphate-buffered saline (PBS) adjusted to a pH = 7.5 with 0.1 M NaOH aqueous solution, under which conditions the carboxylic groups of sodium polyacrylate are ionized to eliminate hydrogen-bonded interactions. The samples and mobile phase were filtered before the measurements using 0.45 µm cellulose acetate membranes (Merck KGaA, Darmstadt, Germany). The specific refractive index increment (*dn*/*dc*) used for the molar mass distribution calculation was determined considering the 100% mass recovery method directly within the SEC-dRI instruments. This method provides an accuracy within 10–20% for copolymers as reported elsewhere [32].

#### 2.5.6. Dynamic Light Scattering (DLS)

The hydrodynamic radius (*R_h_*) was evaluated by DLS using a Nano Zetasizer instrument from Malvern Panalytical Instruments (Westborough, MA, USA), equipped with a helium neon (He–Ne) laser (λ = 633 nm) and a scattering angle of 173°. Measurements were carried out at a temperature of 25.0 ± 0.1 °C. Four independent measurements were performed on each polymer sample.

Initially, a polymer solution (2500 mg L^−1^) was prepared using ultra-pure water and stirred at 400 rpm for 24 h (designated as aggregated (*agg*)). Subsequently, each sample was sonicated for 3 h to disrupt polymer agglomerates in the solution (designated as sonicated (*son*)). Finally, the 2500 mg L^−1^ solution was diluted to 100 mg L^−1^ and filtered through 0.45 µm cellulose acetate membranes (Merck KGaA, Darmstadt, Germany) to remove any remaining agglomerates (designated as filtered (*fil*)). Thus, the hydrodynamic radii determined in this study are defined under these three conditions: *R*_*h*(*agg*)_ (pre-sonication), *R*_*h*(*son*)_ (post-sonication), and *R*_*h*(*fil*)_ (post-dilution and filtration).

#### 2.5.7. Apparent Viscosity and Rheological Measurements

The apparent viscosity of aqueous copolymer solutions was determined using an Anton Paar MCR301 rheometer (Ostfildern, Germany) equipped with a 50 mm diameter plate–plate geometry (PP-50), with a Peltier system (H-PTD200) that maintained a constant temperature of 25.00 ± 0.01 °C throughout the measurements to minimize evaporation. Each 2 mL-sample was subjected to a constant shear rate of 10 s^−1^ for 300 s, followed by a 300 s rest period for thermal equilibration.

After the sample preparation, the first shear-rate sweep was performed, ranging from 1 s^−1^ to 1000 s^−1^, consisting of 31 data points and lasting approximately 80 min. The second rotational test involved five constant shear rate steps of 2 min at 7.34 s^−1^ and 1000 s^−1^, starting at the lowest value. The viscosity of the sample was recorded in all cases. All copolymer solutions used in these experiments had a concentration of 2500 mg L^−1^ and were dissolved in ultra-pure water by stirring at 400 rpm for 24 h.

## 3. Results and Discussion

### 3.1. Synthesis and Spectral Analysis

Immediately following the polymerization reaction, the resulting polymers exhibited markedly different physical characteristics. The product obtained from the aqueous solution appeared as a colorless, viscous fluid, whereas the material synthesized in the pressurized carbon dioxide–ethyl acetate medium contained no apparent residual solvent and presented as a fine, homogeneous powder.

The overall monomer conversion of the synthesized copolymers was determined by analyzing the ^1^H NMR signals corresponding to the vinyl groups relative to the methylene signals of the copolymer backbone. These values are detailed in Table 2, and all required spectra for their calculation are provided in the Supporting Information (Figures S5–S8, Table S1 and Equations ES1–ES4). The results indicate that the monomer double bond conversions ranged from 97 to 99 mol% for all reactions.

Since it is necessary to determine whether a dialysis process is strictly required to obtain a product with acceptable characteristics for its potential future application in enhanced oil recovery (EOR), the majority of the analytical results are presented for both materials obtained: purified solely by precipitation and those purified by both precipitation and dialysis. Hereafter, these samples will be referred to as (1) precipitation and (2) dialysis.

**Figure 2:**
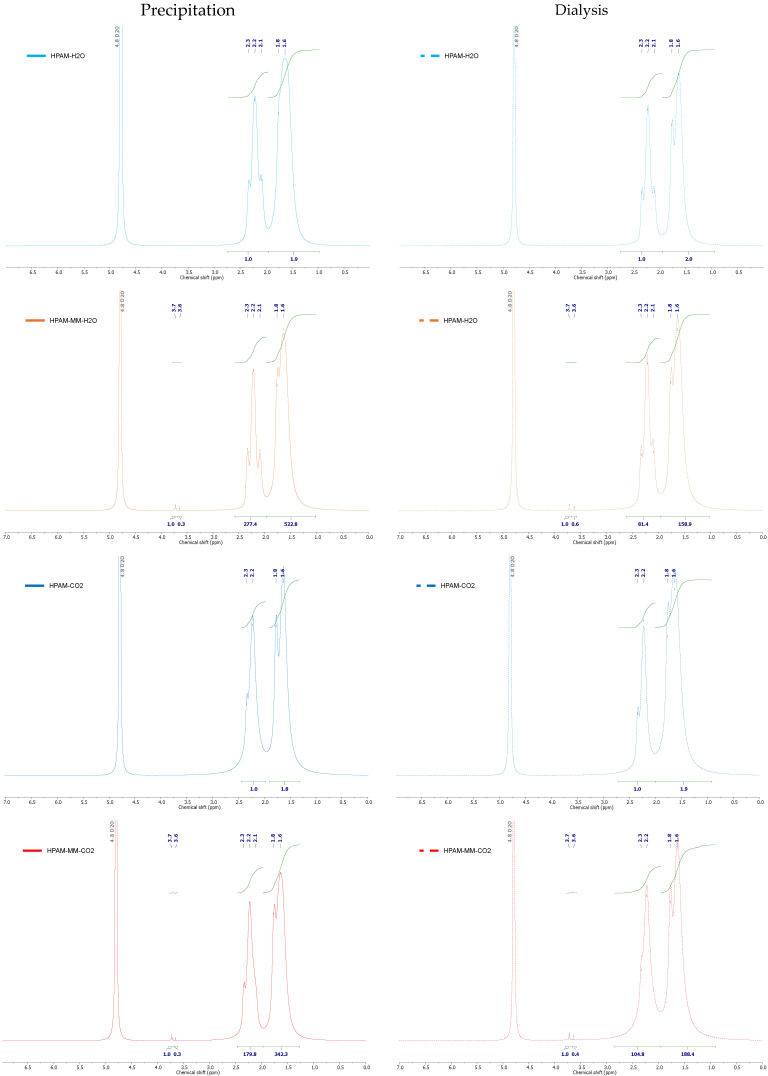
^1^H NMR spectra for the synthesized copolymers, highlighting differences based on the purification method.

^1^H NMR spectra of the synthesized copolymers, comparing both precipitation and dialysis products, are presented in Figure 2. The spectra exhibit characteristic CH_2_ signals between 1.6 and 1.8 ppm, as well as CH signals between 2.1 and 2.4 ppm, which are attributed to the poly(acrylic acid-*co*-acrylamide) backbone [33]. Additionally, HPAM-MM spectra display signals corresponding to the aliphatic chain of PEGMA^500^ between 3.2 and 3.8 ppm (see Supporting Information Table S2). These resonances were also observed in the PEGylated star-shaped macromonomer in earlier studies [21,22].

The incorporation of the star-shaped macromonomer (Boltorn H30–PEGMA^500^–V) into the polymers was determined by ^1^H NMR, adapting the calculation procedure described by Lima et al. [33] (see Supporting Information Equation ES5).

The results, summarized in Table 2, confirm the successful incorporation of the macromonomer, as evidenced by the absence of vinyl signals in the NMR spectra. Furthermore, higher incorporation levels were achieved when the polymers were synthesized in aqueous solution compared to those produced in a pressurized fluid system. A comparison between polymers purified by precipitation and those further purified by dialysis reveals an increased incorporation in the dialysis-purified samples, suggesting that shorter-chain polymers lacking the macromonomer are removed during dialysis while polymers containing the macromonomer are retained within the dialysis bag. This outcome is consistent with the dialysis membrane cutoff being below the macromonomer size, as previously demonstrated for the pure star-shaped macromonomer [21,22]. The absence of vinyl groups in the dialysis product confirms complete polymerization of the macromonomer, a finding that is corroborated by the additional analytical techniques.

^13^C NMR spectroscopy was also performed to further assess the acrylic acid–acrylamide ratio in the synthesized polymers. The results, expressed as mol% of acrylic acid, were derived from the ratio of the integral of the carboxylic acid carbonyl signal to the sum of the integrals of both the carboxylic acid and amide carbonyl signals (detailed spectra, calculations, and equations are provided in the Supporting Information, Figures S9–S16 and Equation ES6). For polymers synthesized in water (Table 2), the presence of the macromonomer did not alter the selectivity between acrylic acid and acrylamide incorporation; the acrylic acid molar percentage in the final products closely matched the targeted composition, with no significant differences observed between the dialyzed and precipitated samples. In contrast, when synthesis was performed in pressurized solvents, a pronounced difference in acrylic acid incorporation was observed between the polymers obtained by precipitation and those purified by dialysis. This outcome is consistent with the limited solubility of acrylamide in the supercritical fluid mixture, which likely leads to the precipitation of polymers with a higher acrylamide content and shorter chain lengths that are subsequently removed during dialysis, thereby yielding a dialysis product enriched in acrylic acid and characterized by longer polymer chains.

**Figure 3:**
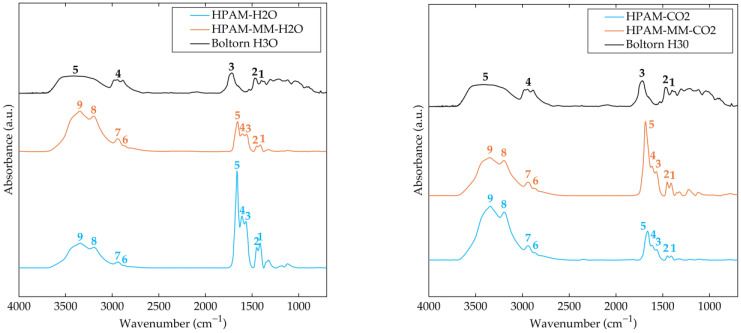
FTIR absorbance spectra of the synthesized copolymers purified by precipitation followed by dialysis, and of Boltorn H30. (**left**) HPAM-H_2_O, HPAM-MM-H_2_O, and Boltorn H30. (**right**) HPAM-CO_2_, HPAM-MM-CO_2_, and Boltorn H30. The numerical labels in the spectra denote the most significant signals observed in the FTIR analysis.

FTIR spectra for the copolymers synthesized in water and for Boltorn H3O are presented in Figure 3. The spectra clearly exhibit the characteristic features of poly(acrylic acid-*co*-acrylamide). Specifically, symmetric and asymmetric N–H stretching bands (signals 8 and 9) are observed between 3000 and 3600 cm^−1^, while the C–H stretching vibrations of CH and CH_2_ groups in the polymer backbone appear as signals 6 and 7. Carbonyl (C=O) stretching vibrations are detected between 1620 and 1720 cm^−1^ (signals 4 and 5). In addition, the bending vibration of the NH_2_ group is evident near 1610 cm^−1^ (signal 4). The deprotonated carboxylic acid is represented by asymmetric and symmetric stretching bands at approximately 1565 and 1410 cm^−1^ (signals 3 and 1), respectively. Moreover, the CH_2_ bending vibration is seen around 1450 cm^−1^, and the C–N stretching vibration in the amide group is observed as signal 2. These assignments are consistent with values reported in the literature [34,35,36].

On the other hand, the FTIR spectrum for Boltorn H3O exhibits the characteristic signals of a hyperbranched polyester. Specifically, it shows a broad O–H stretching band (signals in the region 5 in the figure) between 3200 and 3600 cm^−1^, symmetric and asymmetric stretching bands of CH_2_ and CH_3_ groups (signals in region 4), a carbonyl stretching band (signal 3) near 1730 cm^−1^, and CH_2_ and CH_3_ bending vibrations (signals 2 and 1) at 1470 and 1400 cm^−1^, respectively [37].

In the FTIR spectrum of HPAM-MM-H_2_O, the characteristic signals of Boltorn H30 are not evident, which is not unexpected given that the macromonomer incorporation was below 0.0614 mol% (with Boltorn H3O comprising only 2.75 wt% of the macromonomer). Nonetheless, even at these low levels, the presence of the macromonomer may induce molecular reorganization within the copolymer, thereby enhancing inter- and intrachain interactions—particularly through hydrogen bonding. Such interactions are expected to manifest as shifts in the peak positions of bands associated with hydrogen bonding, such as those corresponding to the amide and carboxylic groups. Kumari et al. [38] demonstrated that FTIR spectroscopy is an effective tool for probing conformational changes, accessibility, and hydrogen bond formation in branched polyesters and polyurethanes, reporting observable shifts in the N–H and C=O bands via deconvolution of overlapping peaks.

**Figure 4:**
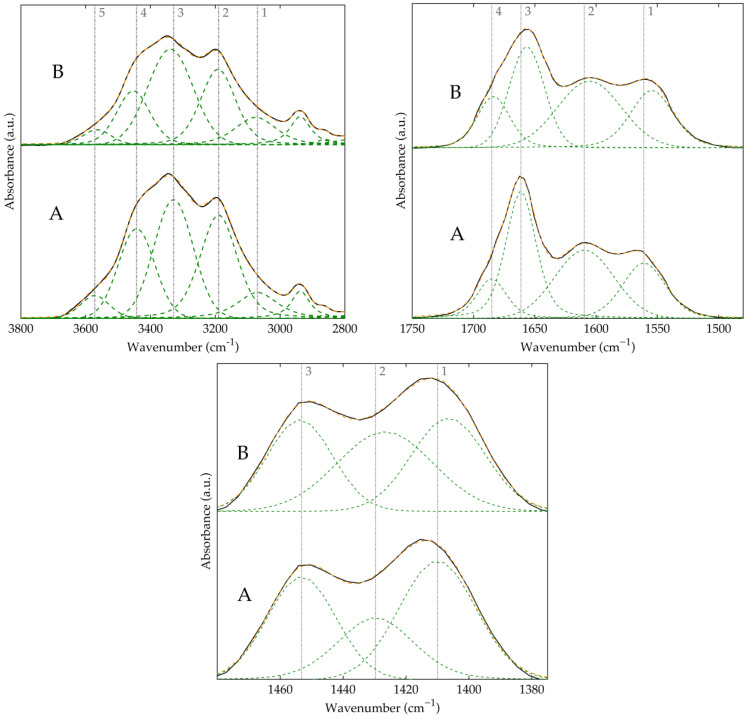
Deconvolution of FTIR regions of HPAM-H2O (A) and HPAM-MM-H2O (B) in the regions: (**top left**) 3800–2800 cm^−1^, (**top right**) 1750–1500 cm^−1^, (**bottom**) 1480–1380 cm^−1^. Original spectrum (black), fitted spectrum (orange), deconvoluted peaks (green). Dashed lines show the position of the maximum for the deconvoluted peaks in sample A for a direct analysis of the shifts observed in the spectra of sample B. The numerical labels next to the dashed lines denote the most significant signals observed in the deconvolution in the FTIR analysis.

Figure 4 presents the deconvoluted FTIR spectra of HPAM-H_2_O and HPAM-MM-H_2_O, highlighting several key regions. In the broad N–H stretching region, distinct signals can be identified: those corresponding to N–H groups hydrogen-bonded with an amide carbonyl (signals 1 and 3), with a carboxylic acid (signals 2 and 4), and free N–H groups (signal 5). Analysis of the carbonyl region reveals that there are no signals corresponding to non-hydrogen-bonded carbonyls (typically found between 1710 and 1740 cm^−1^). Instead, the observed signals indicate that the carbonyl groups in carboxylic acid moieties (signal 4) and in amide groups (signal 3), which are capable of forming medium to strong hydrogen bonds, appear at approximately 1685 and 1660 cm^−1^, respectively. Furthermore, to accurately analyze the C–N stretching vibration, it was necessary to deconvolute the overlapping signals of the symmetric COO^−^ stretching and the C–N stretching in the 1400–1430 cm^−1^ region, as previously reported [36].

Finally, to assess the effect of macromonomer incorporation, we examined the shifts in the previously discussed bands. The N–H stretching bands involved in hydrogen bonding exhibit shifts ranging from 1.6 to 10.1 cm^−1^, whereas the carbonyl bands shift to smaller values between 1 and 6.5 cm^−1^. Additionally, the C–N stretching band shifts by approximately 5 cm^−1^ toward lower wavenumbers, which indicates enhanced hydrogen bonding and increased intramolecular interactions [39]. All corresponding values and spectra for each region and material are reported in Tables S3–S15 and Figures S17–S26 of the Supporting Information.

The results obtained for the copolymers synthesized in the pressurized CO_2_–ethyl acetate system follow similar trends to those observed for the material synthesized in aqueous solution, although these trends are less pronounced. This difference is likely attributable to variations in the acrylamide-to-acrylic acid ratio between materials with and without the macromonomer, as demonstrated by NMR results. As shown in Figure 3, the same characteristic signals observed in all previously discussed copolymers are present. Notably, for the material containing the macromonomer, the carbonyl band appears sharper and is slightly shifted to higher wavenumbers.

**Figure 5:**
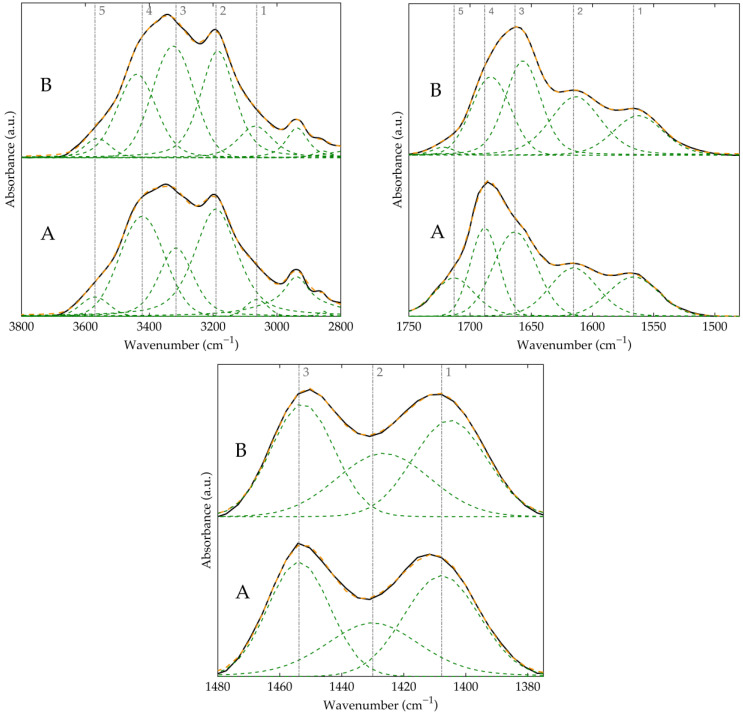
Deconvolution of FTIR regions of HPAM-CO_2_ (A) and HPAM-MM-CO_2_ (B) in the regions: (**top left**) 3800–2800 cm^−1^, (**top right**) 1750–1500 cm^−1^, (**bottom**) 1480–1380 cm^−1^. Original spectrum (black), fitted spectrum (orange), deconvoluted peaks (green). Dashed lines show the position of the maximum for the deconvoluted peaks in sample A for a direct analysis of the shifts observed in the spectra of sample B. The numerical labels next to the dashed lines denote the most significant signals observed in the deconvolution in the FTIR analysis.

Upon deconvolution of the three regions of interest (Figure 5), several differences were observed compared to the polymers synthesized in aqueous solution. The most notable difference is the appearance of a free carbonyl signal, with a maximum at 1713 cm^−1^ for the material without the macromonomer and at 1722 cm^−1^ for the material containing the macromonomer. This signal may be associated with a higher proportion of acrylic acid in the polymer. Additionally, all signals corresponding to carbonyl groups engaged in hydrogen bonding were redshifted by 1.8 to 6.5 cm^−1^, as were the C–N stretching signals. These shifts suggest that the incorporation of the macromonomer in the polymers synthesized in pressurized solvents also induces a structural rearrangement in the polymer, thereby enhancing both intra- and intermolecular hydrogen bonding.

### 3.2. Molar Mass and Molar Mass Distribution

Table 3 summarizes the mass-average molar mass (*Mw*), number-average molar mass (*Mn*), and molar-mass dispersity (*Ð*) of the synthesized copolymers. While high phosphate concentrations are commonly used to suppress copolymer binding in size-exclusion chromatography (SEC) columns, phosphate can also act as a salting-out agent, potentially promoting copolymer adsorption. However, in their dissociated form, dominated by the –COO^−^ group, hydrophobic interactions are suppressed, leading to minimal copolymer adsorption and efficient elution. It is important to note that the effect of phosphate on copolymer behavior in SEC is pH-dependent due to its weak electrolyte nature. As the pH increases, both counterion concentration and ionic strength rise at a constant molar concentration of phosphate, influencing copolymer–column interactions [40]. SEC-MALS-dRI analysis of ionic copolymers can be influenced by factors beyond size exclusion, potentially leading to inaccuracies in *Mw* determination. Despite these potential complexities, both HPAM-MM-H_2_O and HPAM-MM-CO_2_ exhibited increased *Mw* compared to their respective HPAM counterparts, indicating successful incorporation of the macromonomer. The incorporation of Boltorn H30—PEGMA^500^—V measured in the dialysis products resulted in *Mw* values of 4.11 × 10^6^ g mol^−1^ and 5.06 × 10^6^ g mol^−1^ for HPAM-MM-H_2_O and HPAM-MM-CO_2_, respectively. These values are significantly higher than those of the copolymers without macromonomer: HPAM-H_2_O: 3.57 × 10^6^ g mol^−1^, HPAM-CO_2_: 7.96 × 10^6^ g mol^−1^. Similar trends were observed for *Mn*. The *Ð* of HPAM-H_2_O was 1.37, while HPAM-MM-H_2_O exhibited lower *Ð* values (e.g., 1.28 for HPAM-MM-H_2_O), suggesting a more uniform copolymer chain distribution (see Table 3). A similar trend was observed for the precipitation copolymers, with higher *M_w_* and *M_n_*. These observations can be attributed to a loss of smaller chains and/or oligomers during the dialysis purification process. Both, precipitation-purified and dialysis-purified copolymer chromatograms are shown in the Supporting Information Figures S27–S29. The observed variations in *Mw* and *MWD* may be attributed to differences in the intramolecular and intermolecular interactions of the Boltorn H30 core and PEGMA^500^ arms in the macromonomer, leading to diverse incorporation patterns within the copolymer chains, as was previously discussed by Melean et al. [22].

When comparing the different solvents used for the reaction, copolymers synthesized in CO_2_ exhibited higher *Mw* and *Mn* values than those synthesized in water after dialysis. This finding aligns with previous observations by other authors for radical reactions [23,41,42]. These SEC-MALS-dRI results corroborate the previously presented hypothesis by demonstrating that low-molecular-weight fractions are removed during dialysis, suggesting that acrylamide-rich polymers precipitate as low-molecular-weight species during the polymerization in the pressurized CO_2_–ethyl acetate media, and that the incorporation of the macromonomer also leads to the precipitation of copolymers with slightly lower molecular mass.

### 3.3. Structure and Conformational Analysis

Conformational changes of the polymers in solution caused by the incorporation of the star-shaped macromonomer were analyzed by considering the radius of gyration (*R_g_*), measured by static light scattering in the SEC-MALS-dRI measurements, and the hydrodynamic radius (*R_h_*) measured by DLS.

Table 4 presents the mean values of *R_g_* and the filtered hydrodynamic radius (*R*_*h*(*fil*)_) (*R*_*h*(*fil*)_ distributions are provided in the Supporting Information, Figures S30 and S31). An increase in *Rg* was observed for the copolymers containing the star-shaped macromonomer, suggesting that the incorporation of the comonomer introduces steric hindrance that restricts the polymer chain’s coiling ability. At the same time, the elevated *R*_*h*(*fil*)_ values indicate that the PEGMA^500^ arms within the macromonomer enhance copolymer solvation. These findings highlight the intricate interplay between intramolecular interactions and copolymer–solvent interactions in the presented polymer systems.

The conformation and structural configuration of the polymers were assessed by analyzing the calculated ρ values. Poly(acrylic acid-*co*-acrylamide) synthesized in water exhibited a typical random coil conformation [43]. However, when the macromonomer was incorporated—whether in water or in pressurized solvents—ρ decreased to approximately 60% of the value observed for the copolymers without the macromonomer. This reduction indicates that the incorporation of the macromonomer induces a significant change in the polymer structure, where the acrylic acid and acrylamide chains probably arrange around the hyperbranched moieties. Hyperbranched polymers are known for their compact structure, limited flexibility, and low degree of entanglement [44], which further contributes to a more compact polymer conformation. Moreover, as discussed previously, the acrylic acid content in the dialyzed polymers synthesized in pressurized solvents is higher than in those synthesized in water. This compositional difference may also account for the observed conformational variations; for example, a ρ value of 1.45 was measured for HPAM-H_2_O compared to 0.75 for HPAM synthesized in the CO_2_–ethyl acetate system.

To determine whether the incorporation of the macromonomer in the copolymers leads to the formation of stable and extensive intermolecular networks in solution, which in turn increase viscosity, the hydrodynamic radii of the copolymer aggregates were measured at a high concentration (2500 mg L^−1^) before and after sonication. The latter treatment promotes the dissociation of chain-associated aggregates. Table 5 presents the hydrodynamic radius values, *R*_*h*(*agg*)_ and *R*_*h*(*son*)_. The corresponding radius distributions are provided in the Supporting Information, Figures S32 and S33.

Table 5 reveals that the *R*_*h*(*agg*)_ for the copolymers containing the macromonomer is significantly higher than that of poly(acrylic acid-*co*-acrylamide), suggesting the formation of larger and more extensive intermolecular networks or aggregates. Notably, the *R*_*h*(*agg*)_ values for copolymers synthesized in supercritical fluids were generally lower than those synthesized in water. Sonication reduced the *R*_*h*(*son*)_ of the copolymers without the macromonomer, indicating the disruption of interchain networks and the rupture of aggregates. In contrast, copolymers incorporating the star-shaped macromonomer exhibited greater resistance to sonication, suggesting the formation of more robust and stable networks in solution.

A comparison of the *R*_*h*(*son*)_ values obtained after sonication with the previously determined *R*_*h*(*fil*)_ values (see Table 4) at a concentration of 100 mg L^−1^ reveals that the sole procedural difference between the two measurements is the filtration step. For polymers without the macromonomer—where sonication effectively disrupts intermolecular associations—the data indicate that microaggregates remain in solution. These microaggregates are likely removed during filtration, as evidenced by the differences observed between *R*_*h*(*son*)_ and *R*_*h*(*fil*)_ for HPAM-H_2_O (*R*_*h*(*fil*)_: 98.0 nm vs. *R*_*h*(*son*)_: 271.1 nm) and HPAM-CO_2_ samples (*R*_*h*(*fil*)_: 63.8 nm vs. *R*_*h*(*son*)_: 304.2 nm).

### 3.4. Rheological Properties

**Figure 6:**
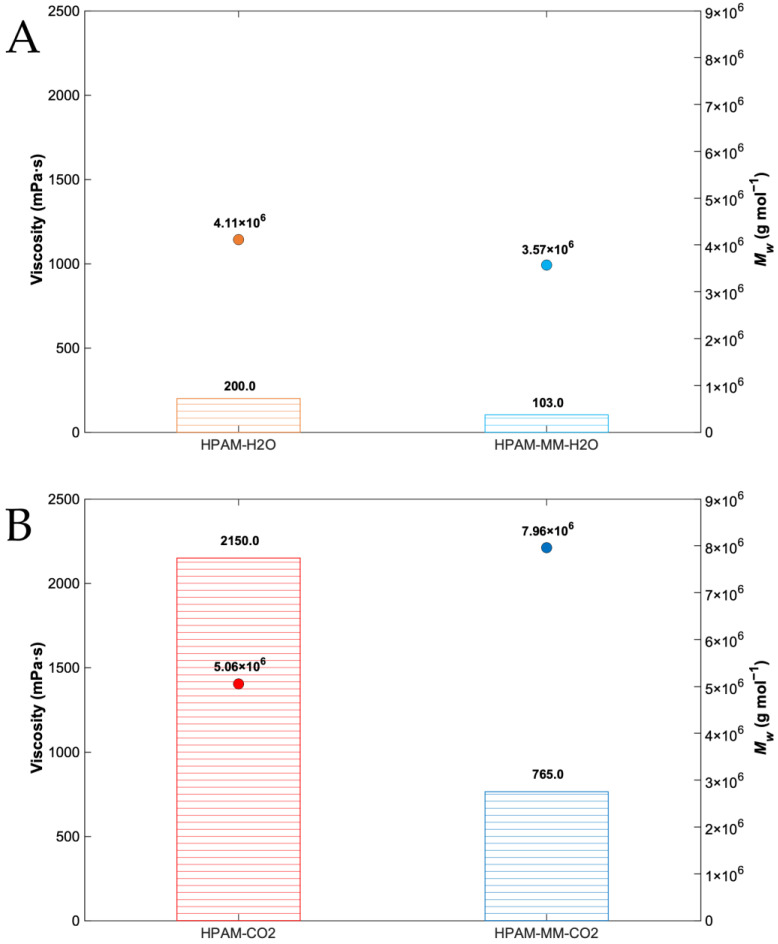
Apparent viscosity measured at 10 s^−1^ for dialyzed copolymer solutions at 2500 mg L^−1^. (**A**) Copolymers synthesized in aqueous solutions. (**B**) Copolymers synthesized in pressurized CO_2_–ethyl acetate mixture.

The viscosity of aqueous copolymer solutions is a critical parameter for enhanced oil recovery applications. Figure 6 compares the viscosities of HPAM-MM and poly(acrylic acid-*co*-acrylamide) solutions, each prepared at 2500 mg L^−1^ in ultra-pure water and measured at a shear rate of 10 s^−1^. These samples include polymers synthesized in aqueous solutions or supercritical fluids and subsequently purified by precipitation followed by dialysis. Notably, all copolymers incorporating the star-shaped macromonomer exhibited significantly higher viscosities than their counterparts. The rheograms, which display the apparent viscosity as a function of shear rate for all copolymer solutions, are provided in the Supporting Information, Figure S34.

Figure 6 demonstrates a substantial disparity in viscosity between copolymers synthesized in supercritical CO_2_ and those prepared in water. Notably, copolymers synthesized in supercritical CO_2_ exhibit viscosities several orders of magnitude greater. This discrepancy can be attributed to several factors, including higher *Mw* and the unique polymerization kinetics in this reaction media, together with the selective precipitation of polymers containing acrylamide and the different ratio of acrylic acid:acrylamide in the polymers. It is most probable that at least two distinct phases (dispersed and continuous) exist within the supercritical CO_2_ reaction media, that explain the multimodal *MWD* of the copolymers. On the other hand, aqueous solution polymerization likely facilitates a more homogeneous and rapid polymerization process, potentially leading to a broad monomodal *MWD* and lower *Mw*, consequently contributing to a lower overall viscosity. Furthermore, the solvent can influence the monomer distribution within the chain, leading to more extended conformations in chains synthesized in CO_2_ compared to those synthesized in water.

HPAM-MM-CO_2_ exhibits the highest viscosity (2150 mPa·s) despite having a molecular weight (5.06 × 10^6^ g·mol^−1^) approximately half that of HPAM-CO_2_ and comparable to HPAM-MM-H_2_O and HPAM-H_2_O. This result confirms the formation of larger and more extensive intermolecular networks or aggregates, as supported by the DLS analysis. These findings highlight the fundamental role of intermolecular interactions in solution viscosity regardless of the molar mass.

### 3.5. Thermal Analysis

The thermal behavior of copolymers, such as poly(acrylic acid-*co*-acrylamide), is complex and involves multiple degradation reactions. Maurer and Harvey [45] combined thermogravimetric analysis coupled with mass spectroscopy (TG-MS) and DSC to study HPAM degradation, demonstrating that acrylamide monomers undergo an initial loss of physically bonded water, followed by imide formation with ammonia release, and subsequent chain scission. Similarly, polyacrylic acid exhibits sequential degradation events: initial extraneous water loss, additional water loss during anhydride formation, and further decomposition of the anhydride accompanied by chain scission. In their DSC analysis, Maurer and Harvey noted that the degradation endotherms, particularly between 200 and 375 °C, were strongly influenced by the degree of acrylic acid neutralization. TG-MS measurements revealed three distinct water-release peaks between 160 and 450 °C, with the peaks at approximately 240 and 280 °C associated with both strongly bound water and acid degradation, alongside ammonia evolution from acrylamide hydrolysis and imide formation near 275 °C. Additionally, Caulfield et al. [46] reviewed degradation pathways in polyacrylamides, confirming that both surface and matrix-bound water are released up to 200 °C—a phenomenon minimized by thorough milling and vacuum drying.

Voronova et al. and Lv et al. [47,48] reported a three-stage degradation process for PAM: water vaporization (25–200 °C), imidization process with subsequent ammonia release (220–335 °C), and chain scission with imide decomposition (>350 °C). Similarly, Van Dyke and Kasperski [49], using TG coupled with GC–FTIR and GC–MS, confirmed that early-stage reactions involve inter- and intramolecular imidization, bicyclic compound formation, crosslinking, and nitrile formation (with CO_2_ release), while major chain breakdown occurs in the later stage. For poly(acrylic acid), Moharram and Khafagi [50,51] identified three distinct steps: decarboxylation (156.8–225.3 °C), anhydride formation (225.3–301 °C), and final degradation of the poly(acrylic anhydride) (301–476.1 °C).

**Figure 7:**
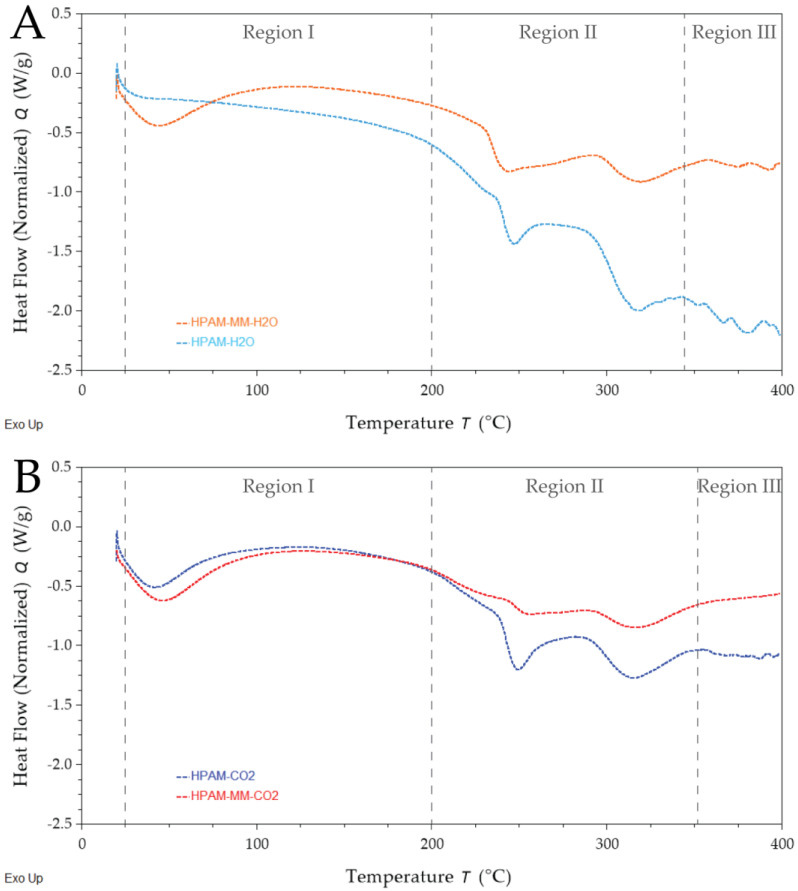
DSC thermograms for polymers purified by precipitation followed by dialysis. (**A**) Copolymers synthesized in aqueous solutions. (**B**) Copolymers synthesized in pressurized CO_2_–ethyl acetate mixture. Regions I, II, and III were identified based on the thermogram of poly(acrylic acid-*co*-acrylamide).

DSC thermograms recorded for the synthesized polymers and presented in Figure 7 can be analyzed considering the three distinct thermal regions.

- Region I (up to ~220 °C): This initial endothermic event is primarily associated with the release of surface and matrix-bound water. Near the upper end of this temperature range, decarboxylation reactions may also occur.
- Region II (up to ~350 °C): Two prominent endothermic peaks are observed in this region. These peaks correspond to the primary chemical modifications of the polymer—namely, cyclization reactions leading to imide and anhydride formation. The energy absorbed during these transitions reflects significant chemical modifications within the polymer.
- Region III (above ~350 °C): In this final stage, further degradation is evident, marked by the breakdown of imide and anhydride groups and chain scission, culminating in the disintegration of the polymer structure.

Table 6 summarizes the key thermal parameters extracted from the DSC thermograms mainly from region II, including the initial, peak, and end temperatures, as well as the associated peak enthalpies. Complete thermograms detailing these transitions are provided in Supporting Information Figures S35 and S36.

A detailed comparison between HPAM-H_2_O and HPAM-MM-H_2_O reveals that the incorporation of the macromonomer shifts the temperature range of Region II. Specifically, the end temperatures of the decomposition peaks increase—from 266.0 °C to 291.7 °C and from 342.5 °C to 359.3 °C, respectively—despite both polymers having similar acrylamide-to-acrylic acid ratios. This shift suggests that the hyperbranched macromonomer disrupts the interaction between amide and carboxylic acid groups during imide and anhydride formation.

For polymers synthesized in supercritical fluids, the trends are less straightforward, likely due to differences of approximately 10% in acrylic acid content and variations in molecular weight distribution—both of which affect thermal behavior. Notably, degradation reactions in Region III were not detectable up to 400 °C in polymers containing the macromonomer, indicating that the steric hindrance introduced by the macromonomer is preserved within the polymer chain. Similar observations were reported by Zhang et al. [52], who compared the thermal degradation of various PAM-type polymers containing N-vinyl pyrrolidone, sulfonated monomers, hydrophobic monomers, or sterically hindered groups. Their study demonstrated that strong interactions between sterically hindering groups and amide groups can inhibit the pyrolysis of amide groups by disrupting intra- and inter-imidization reactions (observed between 220 and 320 °C), thereby enhancing the thermal stability of the polymer. The remaining sterically hindered amide groups underwent pyrolysis only at temperatures above 320 °C.

### 3.6. Morphological Analysis

**Figure 8:**
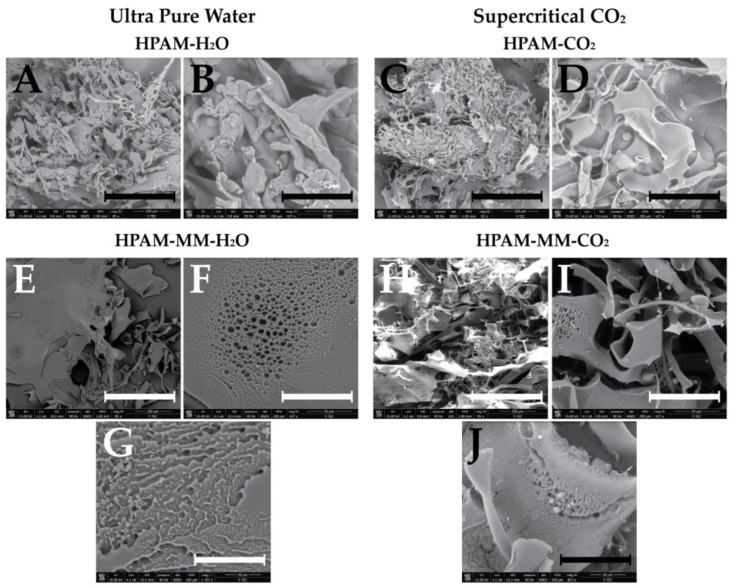
SEM micrographs for the synthesized and purified (by precipitation + dialysis) copolymers. Scale bars: (400 µm for (**A**,**C**,**E**,**H**); 100 µm for (**B**,**D**,**F**,**I**); 40 µm for (**G**,**J**)).

Figure 8 presents the scanning electron microscopy micrographs of the synthesized copolymers following dialysis and lyophilization. Figure 8A,B illustrate the morphology of poly(acrylic acid-*co*-acrylamide) prepared in aqueous solution at 85× and 427× magnifications, respectively, revealing irregular structures with smooth surfaces. Figure 8C,D display the same copolymer but synthesized in supercritical fluids at the same magnifications, with no significant morphological differences observed compared to the aqueous system. Figure 8E–G correspond to the polymer containing the star-shaped macromonomer (HPAM-MM) synthesized in aqueous solution, captured at 85×, 427×, and 1707× magnifications, respectively. These images reveal the presence of small surface pores, with diameters ranging from approximately 4 to 10 µm. Similarly, Figure 8H–J depict HPAM-MM synthesized in supercritical fluids at the corresponding magnifications, where comparable superficial pores are observed. This observation suggests that the incorporation of the star-shaped macromonomer promotes the formation of these morphological features.

Moreover, while poly(acrylic acid-*co*-acrylamide) exhibited smooth surfaces irrespective of the synthesis medium, the HPAM-MM copolymers displayed distinct porosity along their surfaces, indicating that the inclusion of the macromonomer induces significant, observable macroscopic changes. Notably, no substantial differences were observed between HPAM-MM copolymers synthesized in water and those produced in supercritical fluids.

### 3.7. Purification Process

Previous results indicate that the purification process directly influences the final properties of the polymer solution, particularly for polymers synthesized in supercritical fluid media. As confirmed by SEC-MALS-dRI analysis, dialysis yields polymers with higher molecular weights and narrower molecular weight distributions.

Although NMR spectroscopy showed no significant difference in the acrylic acid-to-acrylamide ratio for copolymers synthesized with the macromonomer in aqueous solutions, copolymers prepared in a pressurized CO_2_–ethyl acetate medium with molecular sizes exceeding 14 kDa exhibited a 40–70% increase in acrylic acid content compared to those synthesized in water. Consequently, dialysis appears essential to ensure reproducibility and a more homogeneous product when employing supercritical solvents such as CO_2_ and ethyl acetate.

Nevertheless, a mass balance assessment remains necessary to evaluate the dialysis purification process. Table 7 presents the percentage of mass lost during dialysis for each synthesized polymer. Notably, copolymers synthesized in supercritical CO_2_ exhibit a greater mass reduction than those prepared in water. For example, the purified mass recovery for the HPAM-H_2_O sample is 37.3%, compared to 26.5% for HPAM-CO_2_. Moreover, a substantial reduction in purified mass is observed when comparing polymers containing the macromonomer with poly(acrylic acid-*co*-acrylamide). Specifically, the HPAM-MM-CO_2_ sample yielded only 8.1% purified mass, in contrast to 26.5% for HPAM-CO_2_.

These results indicate that a significant fraction of the copolymers comprises low-molar-mass species, which are removed during the dialysis purification process. In the aqueous system, these findings are consistent with those reported by Kuldasheva et al. [53] for poly(acrylic acid-*co*-acrylamide) synthesized at temperatures above 55 °C using the same initiator as in our study. For the copolymers synthesized in pressurized solvents, the reduced solubility—resulting from the incorporation of acrylamide and the macromonomer—leads to a higher precipitation of short-chain polymers during polymerization, which in turn contributes to a lower recovered mass after dialysis.

## 4. Conclusions

Four copolymers were synthesized using different reaction media: (i) poly(acrylic acid-*co*-acrylamide) in aqueous solution, (ii) poly(acrylic acid-*co*-acrylamide) in a high-pressure CO_2_–ethyl acetate mixture, (iii) a copolymer composed of acrylic acid, acrylamide, and a designed star-shaped hyperbranched macromonomer in aqueous solution, and (iv) the same macromonomer-containing copolymer synthesized in a high-pressure CO_2_–ethyl acetate mixture.

Comprehensive characterization by spectroscopic and chromatographic techniques confirmed the successful incorporation of the macromonomer into the polymer chain and elucidated variations in the molecular structure and solution conformation. Copolymers incorporating the macromonomer at levels ranging from 0.022 to 0.061 mol% were obtained, exhibiting higher hydrodynamic radii, increased radii of gyration, and enhanced thickening properties compared to those lacking the macromonomer. FTIR spectroscopy showed that the incorporation of the macromonomer promotes hydrogen bonding within the polymers. Microscopic images further revealed distinct porosity in the polymer surface as a direct consequence of macromonomer incorporation. Rotational rheometric analysis of the copolymer solutions revealed a significant increase in viscosity, which can be attributed to the associative effects of the polymer chains and the promotion of interchain networks or aggregates in aqueous solutions. Contrary to the common assumption that higher molar mass directly correlates with increased viscosity, the present findings indicate that factors such as polymer architecture and interchain interactions have a more pronounced effect. The substantial viscosity enhancement observed for HPAM-MM copolymers is primarily attributed to the formation of extensive interchain networks facilitated by the hyperbranched macromonomer, rather than simply an increase in molar mass.

This study also reports, for the first time, the synthesis of a copolymer composed of acrylic acid, acrylamide, and a hyperbranched macromonomer using a high-pressure solvent mixture. Notably, this copolymer exhibited the highest viscosity in aqueous solution among all the polymers examined.

Additionally, the purification methods, precipitation alone versus precipitation followed by dialysis, were evaluated to determine whether the more challenging dialysis process is necessary to obtain high-quality, reproducible polymer characteristics. During dialysis, low-molecular-weight fractions enriched in acrylamide are removed, while high-purity, high-average-molar-mass polymers are recovered. However, mass balance analysis revealed that only a small fraction of the synthesized product is retained following purification. Moreover, the requirement for an additional drying step (e.g., lyophilization) could substantially increase the overall production cost.

## Figures and Tables

**Table 1:** Experimental conditions for the synthesis of HPAM-MM-H_2_O, HPAM-MM-CO_2_, HPAM-H_2_O, and HPAM-CO_2_ copolymers.

Copolymer	Boltorn H30—PEGMA^500^—V (g)	Boltorn H30—PEGMA^500^—V Content Relative to the Total Weight of Monomers in the Feed (wt%)	AAm (g)	AAm (mol)	AAm (%mol)	AAc (g)	AAc (mol)	AAc (%mol)	Initiator (%mol) (×10^−2^)	Temperature (°C)	Initial Pressure (bar)
HPAM-H_2_O		-	10.6795	0.15	72.92	4.0198	0.05	27.08	3.08	65	-
HPAM-MM-H_2_O	0.1865	0.85	10.6895	0.15	73.32	3.9415	0.05	26.66	3.01	65	-
HPAM-CO_2_		-	10.7187	0.15	74.99	3.6242	0.05	25.01	3.23	90	320
HPAM-MM-CO_2_	0.1723	0.85	10.7174	0.15	74.92	3.6345	0.05	25.06	3.23	90	320

**Table 2:** HPAMs and HPAM-MMs copolymer characterization by NMR: Global residual monomer fraction, global monomer conversion, molar percentage of acrylic acid, and Boltorn H30—PEGMA^500^ incorporation in the synthesized copolymers.

Copolymer	Global Residual Monomers Fraction (mol%) ^a^	Global Monomer Conversion (mol%) ^a^	AAc Precipitation (mol%) ^b^	AAc Dialysis (mol%) ^b^	Boltorn H30—PEGMA^500^–V Incorporation (Precipitation) (mol%)	Boltorn H30—PEGMA^500^–V Incorporation (Dialysis) (mol%)
HPAM-H_2_O	1.5	98.5	23.3	24.4	-	-
HPAM-MM-H_2_O	0.9	99.1	23.6	23.3	0.0220 ± 0.0005	0.0614 ± 0.0005
HPAM-CO_2_	2.7	97.3	24.3	41.4	-	-
HPAM-MM-CO_2_	0.5	99.5	20.0	33.4	0.0278 ± 0.0005	0.0477 ± 0.0005

^a^ Obtained from ^1^H NMR before purification. ^b^ Obtained from ^13^C NMR.

**Table 3:** Mass-average molar masses (*M_w_*), number-average molar numbers (*M_n_*), and molar-mass dispersity (*Ð*) of the synthesized copolymers after purification.

Sample	Precipitation			Dialysis		
	*Mw* (g mol^−1^) (×10^6^)	*M_n_* (g mol^−1^) (×10^6^)	*Ð*	*M_w_* (g mol^−1^) (×10^6^)	*M_n_* (g mol^−1^) (×10^6^)	*Ð*
HPAM-H_2_O	2.54 ± 0.03	1.47 ± 0.03	1.72	3.57± 0.04	2.60 ± 0.04	1.37
HPAM-MM-H_2_O	3.52 ± 0.04	2.78 ± 0.05	1.27	4.11 ± 0.04	3.20 ± 0.05	1.28
HPAM-CO_2_	0.96 ± 0.01	0.39 ± 0.01	2.47	7.96 ± 0.06	7.24 ± 0.07	1.10
HPAM-MM-CO_2_	0.86 ± 0.01	0.32 ± 0.01	2.68	5.06 ± 0.04	3.52 ± 0.06	1.44
MM	-	-	-	0.14 ± 0.01	0.06 ± 0.01	2.44

**Table 4:** Radius of gyration (*R_g_*), hydrodynamic radius (*R*_*h*(*fil*)_), and *R_g_*/*R*_*h*(*fil*)_ ratio (***ρ***) of the synthesized copolymers after purification (concentration: 100 mg L^−1^).

Dialyzed Copolymer	*R_g_* (nm)	*R*_*h*(*fil*)_ (nm)	*ρ* (*R*_*g*_/*R*_*h*(*fil*)_)	Conformation
MM	5.2 ± 0.1	-	-	-
HPAM-H_2_O	141.9 ± 0.7	98 ± 3	1.45	Random coil
HPAM-MM-H_2_O	148.8 ± 0.7	178 ± 2	0.84	Compact sphere
HPAM-CO_2_	47.8 ± 0.3	64 ± 1	0.75	Compact sphere
HPAM-MM-CO_2_	60.0 ± 0.4	143 ± 4	0.42	Highly compact structures

**Table 5:** Hydrodynamic radius of aggregates (*R*_*h*(*agg*)_) and hydrodynamic radius after sonication (*R*_*h*(*son*)_) of the purified synthesized copolymers (concentration: 2500 mg L^−1^).

Copolymer	*R*_*h*(*agg*)_ (nm)	*R*_*h*(*son*)_ (nm)
HPAM-H_2_O	2243 ± 98	271 ± 24
HPAM-MM-H_2_O	2493 ± 65	2304 ± 57
HPAM-CO_2_	1670 ± 92	304 ± 20
HPAM-MM-CO_2_	2468 ± 64	2046 ± 82

**Table 6:** Peak analysis from the DSC thermograms for the copolymers purified by precipitation followed by dialysis.

	HPAM-H_2_O	HPAM-MM-H_2_O	HPAM-CO_2_	HPAM-MM-CO_2_
Initial Temperature (°C)	200.0	208.0	192.8	190.0
Maximum Temperature (°C)	245.6	241.8	248.4	253.7
Final Temperature (°C)	266.0	291.7	283.5	289.4
Peak Enthalpy (J·g^−1^)	76.3	118.1	107.6	44.0
Initial Temperature (°C)	282.9	291.7	285.5	289.4
Maximum Temperature (°C)	311.3	319.1	314.8	360.0
Final Temperature (°C)	342.5	359.3	353.2	316.5
Peak Enthalpy (J·g^−1^)	108.9	92.9	113.7	58.4
Initial Temperature (°C)	357.0	359.3	355.0	-
Maximum Temperature (°C)	379.7	392.3	388.2	-
Final Temperature (°C)	389.8	399.0	392.4	-
Peak Enthalpy (J·g^−1^)	23.6	13.9	9.11	-

**Table 7:** Mass yield of the dialysis purification process of copolymers.

Copolymer	Lost Mass (%)	Purified Mass (%)
HPAM-H_2_O	62.7	37.3
HPAM-MM-H_2_O	87.6	12.4
HPAM-CO_2_	73.5	26.5
HPAM-MM-CO_2_	91.9	8.1

## Data Availability

The original contributions presented in this study are included in the article/Supplementary Materials. Further inquiries can be directed to the corresponding author.

## Supplementary Materials

The following supporting information can be downloaded at https://www.mdpi.com/article/10.3390/polym17070964/s1. Figure S1. Chemical structure of Boltorn H30; Figure S2. Chemical structure of Boltorn H30-PEGMA^500^-V (MM); Figure S3. Chemical structure of poly(acrylamide-*co*-acrylic acid) copolymer (HPAM); Figure S4. Chemical structure of poly(acrylamide-*co*-acrylic acid) copolymer modified with MM (HPAM-MM); Figure S5. ^1^H NMR of copolymer HPAM-H_2_O before purification; Figure S6. ^1^H NMR of copolymer HPAM-MM-H_2_O before purification; Figure S7. ^1^H NMR of copolymer HPAM-CO_2_ before purification; Figure S8. ^1^H NMR of copolymer HPAM-MM-CO_2_ before purification; Table S1. Assignation of ^1^H NMR of copolymer HPAM and HPAM-MM synthetized before purification; Equation ES1: % Global residual monomers fraction in HPAM; Equation ES2: % Global monomer conversion in HPAM; Equation ES3: % Global residual monomers fraction in HPAM-MM; Equation ES4: % Global monomer conversion in HPAM-MM; Table S2. Assignation of ^1^H NMR of copolymer HPAM and HPAM-MM synthetized after purification; Equation ES5: % PEGMA^500^ in the copolymer structure; Figure S9. ^13^C NMR of copolymer HPAM-H_2_O-Precipitate; Figure S10. ^13^C NMR of copolymer HPAM-H_2_O-Dialyzate; Figure S11. ^13^C NMR of copolymer HPAM-MM-H_2_O-Precipitate; Figure S12. ^13^C NMR of copolymer HPAM-MM-H_2_O-Dialyzate; Figure S13. ^13^C NMR of copolymer HPAM-CO_2_-Precipitate; Figure S14. ^13^C NMR of copolymer HPAM-CO_2_-Dialyzate; Figure S15. ^13^C NMR of copolymer HPAM-MM-CO_2_-Precipitate; Figure S16. ^13^C NMR of copolymer HPAM-MM-CO_2_-Dialyzate; Equation ES6: % Acrylic acid in the copolymer structure; Figure S17. Deconvolution procedure for HPAM-H_2_O in the three relevant regions: N-H stretching region between 3800 and 2600 cm^−1^ (first column), carbonyl region between 1800 and 1500 cm^−1^ (second column), and C-N stretching region 1375 and 1485 cm^−1^ (third column). Original spectrum (black), Fitted spectrum (orange), deconvoluted peaks (green); Figure S18. FTIR Absorbance spectrum of HPAM-H_2_O; Table S3. Peak deconvolution of HPAM-H_2_O in the region 3800–2600 cm^−1^; Table S4. Peak deconvolution of HPAM-H_2_O in the region 1750–1480 cm^−1^; Table S5. Peak deconvolution of HPAM-H_2_O in the region 1485–1375 cm^−1^; Table S6. Peak deconvolution of HPAM-H_2_O in the region 1485–1375 cm^−1^ forcing a hidden peak in the region 1405–1410 cm^−1^; Figure S19. Peak deconvolution of HPAM-H_2_O in the region 1485–1375 cm^−1^ forcing a hidden peak in the region 1405–1410 cm^−1^; Figure S20. Peak deconvolution of HPAM-H_2_O in the three regions N-H stretching region between 3800 and 2600 cm^−1^ (top left), carbonyl region between 1800 and 1500 cm^−1^ (top right), and the C-N stretching region 1375 and 1485 cm^−1^ (bottom). Original spectrum (black), Fitted spectrum (orange), deconvoluted peaks (green); Figure S21. FTIR Absorbance spectrum of HPAM-MM-H_2_O; Table S7. Peak deconvolution of HPAM-MM-H_2_O in the region 3800–2600 cm^−1^; Table S8. Peak deconvolution of HPAM-MM-H_2_O in the region 1750–1480 cm^−1^; Table S9. Peak deconvolution of HPAM-MM-H_2_O in the region 1485–1375 cm^−1^ forcing a hidden peak in the region 1405–1410 cm^−1^; Figure S22. Peak deconvolution of HPAM-MM-H_2_O in the three regions N-H stretching region between 3800 and 2600 cm^−1^ (top left), carbonyl region between 1800 and 1500 cm^−1^ (top right), and the C-N stretching region 1375 and 1485 cm^−1^ (bottom). Original spectrum (black), Fitted spectrum (orange), deconvoluted peaks (green); Figure S23. FTIR Absorbance spectrum of HPAM-CO_2_; Table S10. Peak deconvolution of HPAM-CO_2_ in the region 3800–2600 cm^−1^; Table S11. Peak deconvolution of HPAM-CO_2_ in the region 1750–1480 cm^−1^; Table S12. Peak deconvolution of HPAM-CO_2_ in the region 1485–1375 cm^−1^forcing a hidden peak in the region 1405–1410 cm^−1^; Figure S24. Peak based deconvolution of HPAM-CO_2_ in the three regions: N-H stretching region between 3800 and 2600 cm^−1^ (top left), carbonyl region between 1800 and 1500 cm^−1^ (top right), and the C-N stretching region 1375 and 1485 cm^−1^ (bottom). Original spectrum (black), Fitted spectrum (orange), deconvoluted peaks (green); Figure S25. FTIR Absorbance spectrum of HPAM-MM-CO_2_; Table S13. Peak deconvolution of HPAM-MM-CO_2_ in the region 3800–2600 cm^−1^; Table S14. Peak deconvolution of HPAM-MM-CO_2_ in the region 1750–1480 cm^−1^; Table S15. Peak deconvolution of HPAM-MM-CO_2_ in the region 1485–1375 cm^−1^ forcing a hidden peak in the region 1405–1410 cm^−1^; Figure S26. Peak deconvolution of HPAM-MM-CO_2_ in the regions: N-H stretching region between 3800 and 2600 cm^−1^ (top left), carbonyl region between 1800 and 1500 cm^−1^ (top right), and the C-N stretching region 1375 and 1485 cm^−1^ (bottom). Original spectrum (black), Fitted spectrum (orange), deconvoluted peaks (green); Figure S27. SEC-MALS-dRI: Differential molar mass distribution of synthesized copolymers studied by precipitation purification method; Figure S28. SEC-MALS-dRI: Differential molar mass distribution of synthesized copolymers studied by dialysate purification method; Figure S29. SEC-MALS-dRI: Differential molar mass distribution of MM; Figure S30. DLS intensity-based hydrodynamic radius analysis of the synthesized copolymers in ultrapure water filters after purification for precipitation. [Copolymer]=100 mg L^−1^. Temperature: 25 °C; Figure S31. DLS intensity-based hydrodynamic radius analysis of the synthesized copolymers in ultrapure water filters after purification for dialysis. [Copolymer]=100 mg L^−1^. Temperature: 25 °C; Figure S32. DLS intensity-based hydrodynamic radius analysis of the synthesized copolymers in ultrapure water aggregate after purification for dialysate. [Copolymer]=2500 mg L^−1^. Temperature: 25 °C; Figure S33. DLS intensity-based hydrodynamic radius analysis of the synthesized copolymers in ultrapure water sonicate after purification for dialysate. [Copolymer]=2500 mg L^−1^. Temperature: 25 °C; Figure S34. Apparent viscosity of synthetized copolymers in ultra-pure water after purification for dialysate. [Copolymer]=2500 mg L^−1^, temperature: 25 °C; Figure S35. DSC thermograms analyzed of synthetized copolymers in aqueous solutions; Figure S36. DSC thermograms analyzed of synthetized copolymers in pressurized CO_2_-ethyl acetate mixture.
