# Supplementary material for: A Comparative Study of Two Synthesis Methods for Poly(Acrylic Acid-Co-Acrylamide) Incorporating a Hyperbranched Star-Shaped Monomer
Source: Polymers (Basel). 2025 Apr 1;17(7):964. doi: 10.3390/polym17070964 (PMC11991247; doi:10.3390/polym17070964)
Supplement: Supplementary file 1 [file polymers-17-00964-s001.zip › polymers-3482012-supplementary.pdf]

Supporting Information

A Comparative Study of Two Synthesis Methods for Poly(acrylic acid-*co*-acrylamide) Incorporating a Hyper-branched Star-Shaped Monomer

Ramses S. Meleán Brito[1,2], Agustín Iborra[3], Juan M. Padró[3,4], Cristian Villa-Pérez[3,4], Miriam C. Strumia[1,2], Facundo Mattea[1,2], Juan M. Giussi[3,4] and Juan M. Milanesio[2,5*]

[1] Universidad Nacional de Córdoba, Facultad de Ciencias Químicas, Departamento de Química Orgánica. Av. Haya de la Torre y Av. Medina Allende, Córdoba, X5000HUA, Argentina.

[2] CONICET, Instituto de Investigación y Desarrollo en Ingeniería de Procesos y Química Aplicada (IPQA – UNC – CONICET). Av. Vélez Sarsfield 1611, Córdoba, X5016GCA, Argentina.

[3] YPF TECNOLOGÍA S. A., Av. Del Petróleo s/n (entre 129 y 143), Berisso, B1923, Argentina

[4] Departamento de Química, Facultad de Ciencias Exactas, UNLP, 47 and 115, La Plata, B1900AJL, Argentina

[5] Universidad Nacional de Córdoba, Facultad de Ciencias Exactas, Físicas y Naturales. Departamento de Química Industrial y Aplicada. Av. Vélez Sarsfield 1611, Córdoba, X5016GCA, Argentina.

Correspondence to: juan.milanesio@unc.edu.ar

Supporting Information

The chemical structure of the compounds

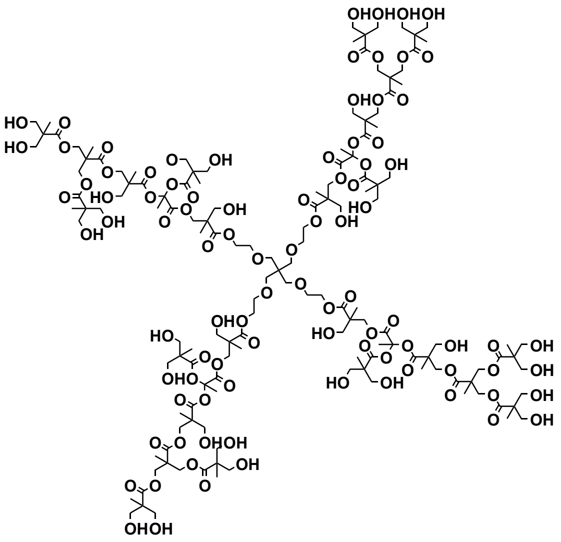

Boltorn H30

Figure S1. Chemical structure of Boltorn H30.

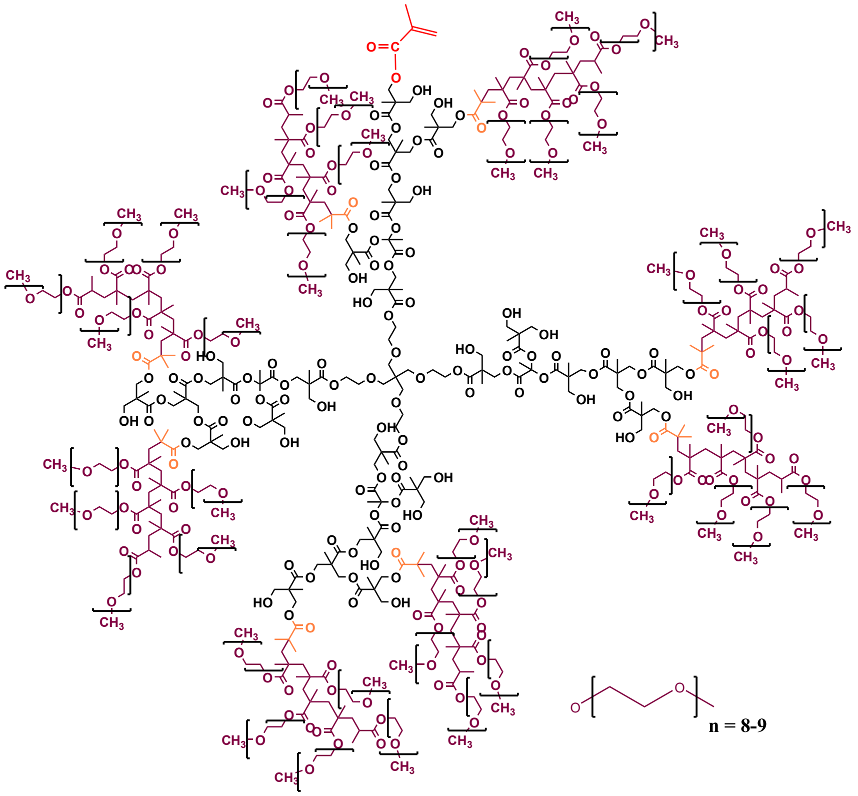

Figure S2. Chemical structure of Boltorn H30-PEGMA[500]-V (MM).

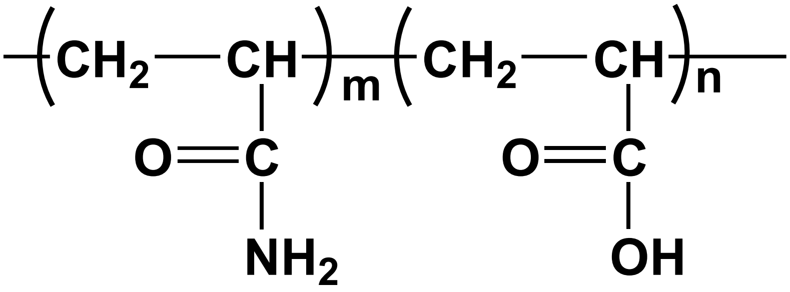

Figure S3. Chemical structure of poly(acrylamide-*co*-acrylic acid) copolymer (HPAM).

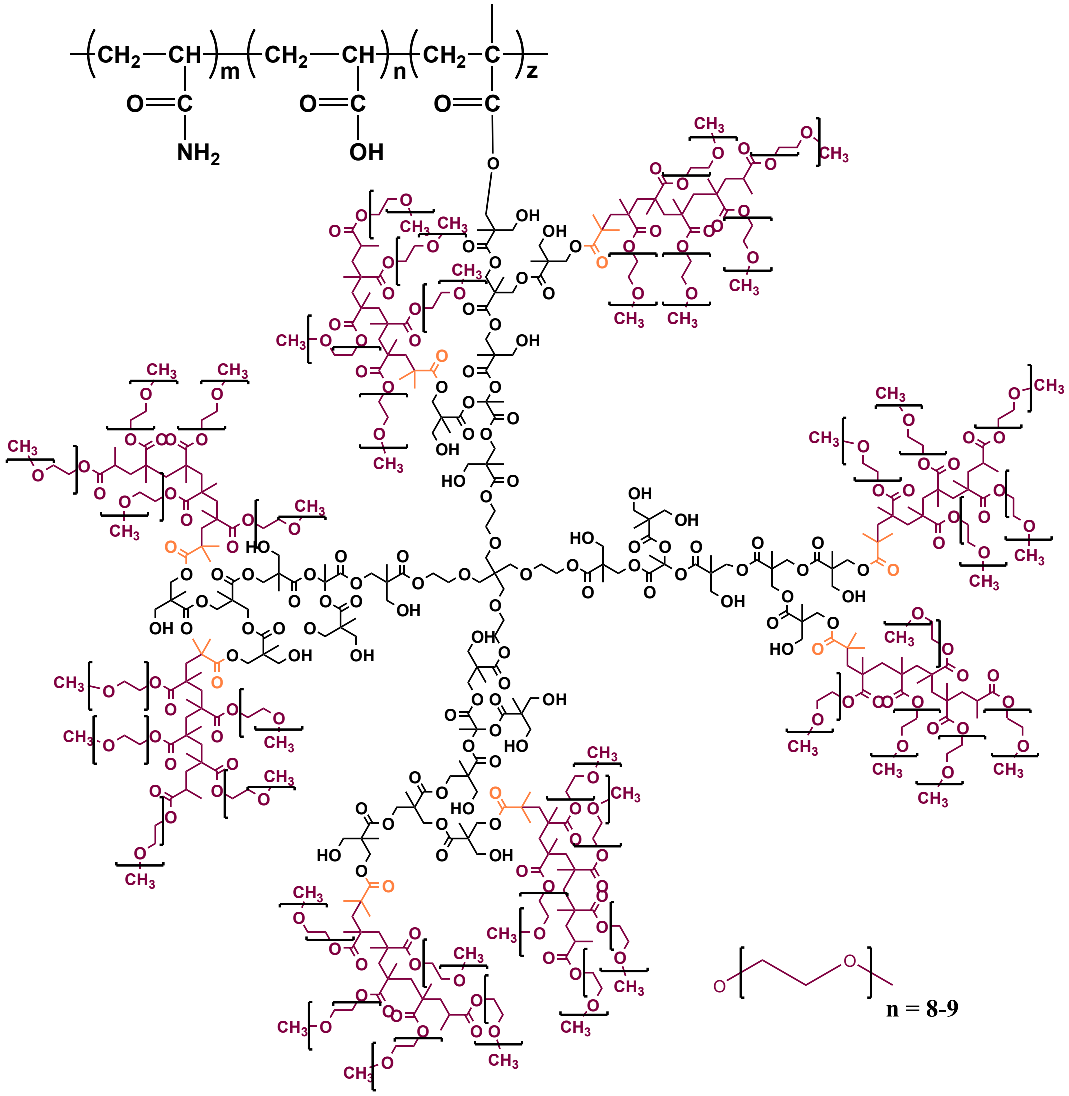

Figure S4. Chemical structure of poly(acrylamide-*co*-acrylic acid) copolymer modified with MM (HPAM-MM).

^{1}H NMR and ^{13}C NMR spectroscopy of the synthesized copolymers before and after purification

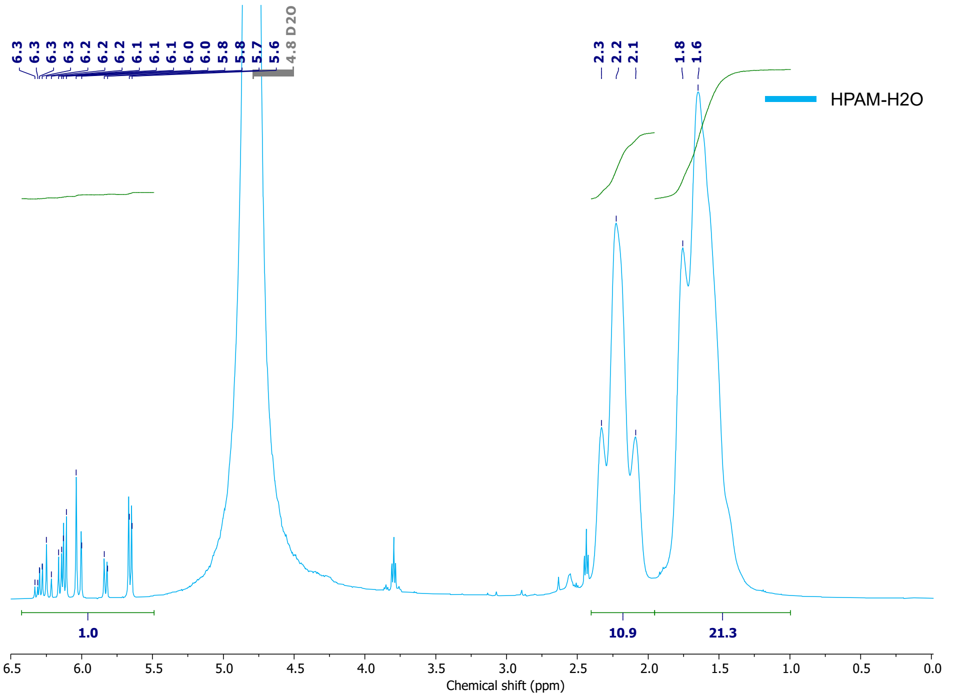

Figure S5. ^{1}H NMR of copolymer HPAM-H_2O before purification.

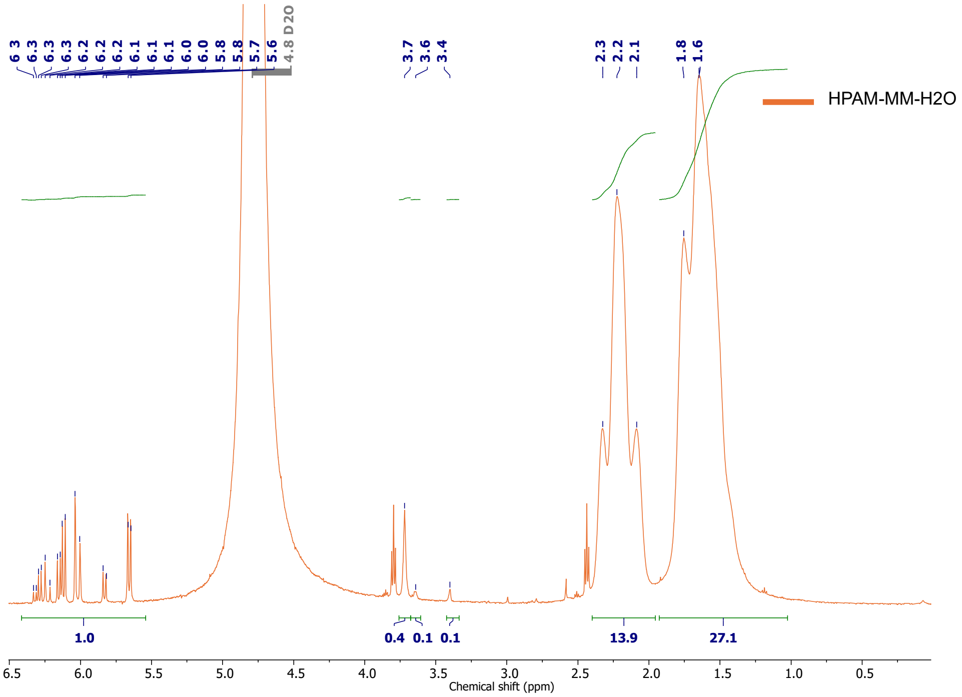

Figure S6. ^{1}H NMR of copolymer HPAM-MM-H_2O before purification.

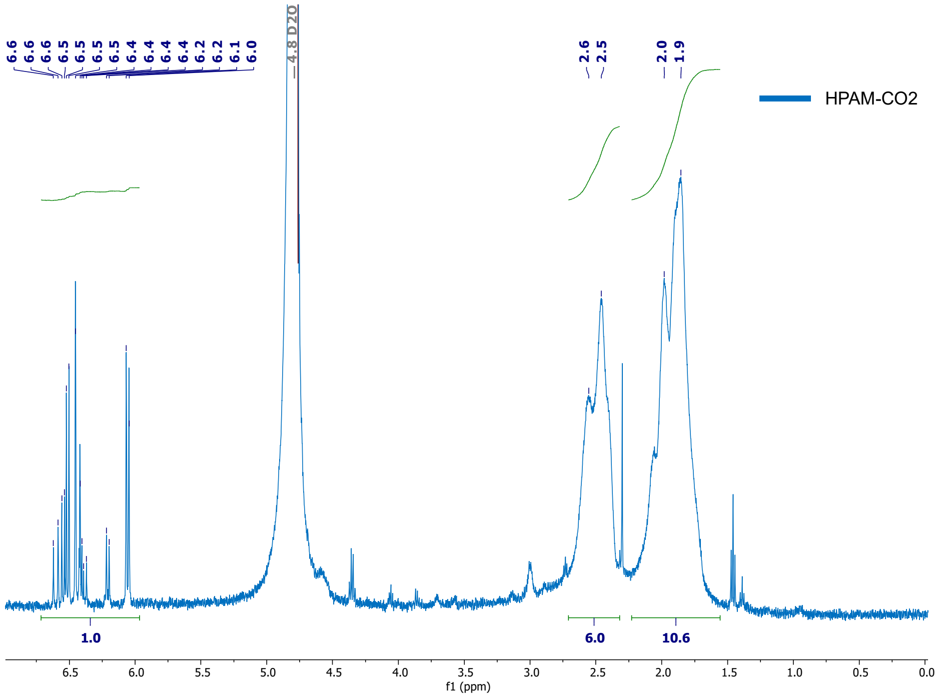

Figure S7. ^{1}H NMR of copolymer HPAM-CO_2 before purification

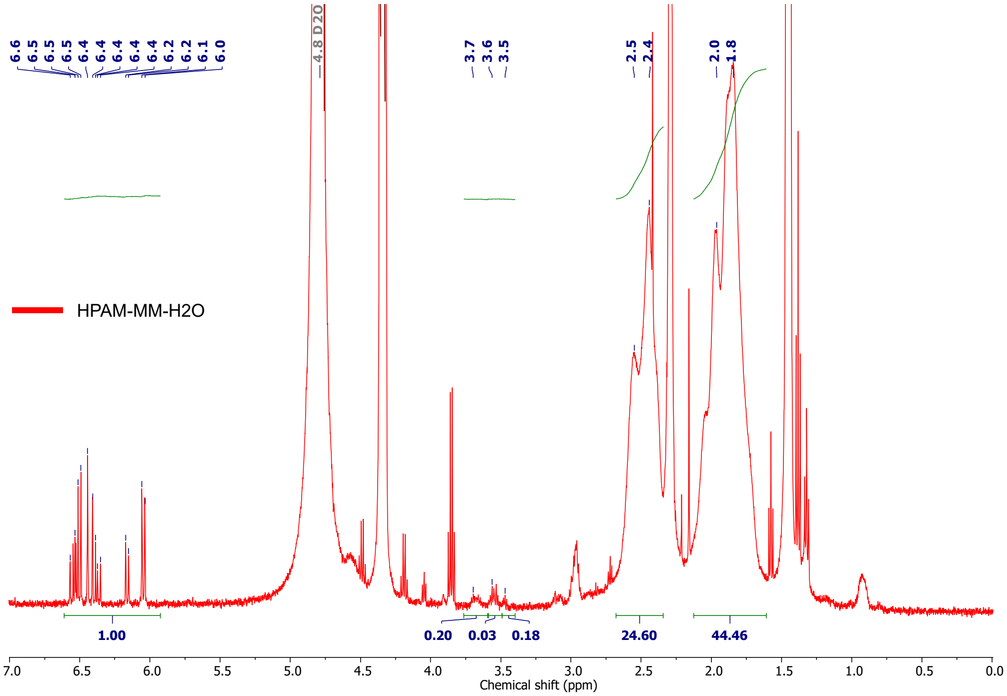

Figure S8. ^{1}H NMR of copolymer HPAM-MM-CO_2 before purification.

Table S1. Assignation of ^{1}H NMR of copolymer HPAM and HPAM-MM synthetized before purification.

Signal (ppm)	Multiplicity	Assignation
≈ 1.6 – 2.0	Multiplet	-C**H**$_2$ (Polymer signal)
≈ 2.1 – 2.5	Multiplet	-C**H** (Polymer signal)
≈ 3.5 – 3.7	Multiplet	-C**H**$_2$ (PEGMA^{500} signal)
≈ 5.6 – 6.6	Multiplet	=C**H**$_2$, and =C**H** (Vinyl groups signal)

Equation ES1: % Global residual monomers fraction in HPAM

$$\% \, Global\ fraction\ in\ residual\ monomers = \frac{\frac{Vinyl\ signal\ (=CH)\ (5.6-6.6\ ppm)}{6}}{Polyimeric\ signal\ (-CH)\ (2.1-2.5\ ppm) + \frac{Vinyl\ signal\ (=CH)\ (5.6-6.6\ ppm)}{6}} * 100$$

Equation ES2: % Global monomer conversion in HPAM

$$\% \, Global\ monomer\ convertion = \frac{Polyimeric\ signal\ (-CH)\ (2.1-2.5\ ppm)}{Polyimeric\ signal\ (-CH)\ (2.1-2.5\ ppm) + \frac{Vinyl\ signal\ (=CH)\ (5.6-6.6\ ppm)}{6}} * 100$$

Equation ES3: % Global residual monomers fraction in HPAM-MM

$$\% \, Global\ fraction\ in\ residual\ monomers = \frac{\frac{Vinyl\ signal\ (=CH)\ (5.6-6.6\ ppm)}{8}}{Polyimeric\ signal\ (-CH)\ (2.1-2.5\ ppm) + \frac{PEGMA^{500}\ signal\ (-CH_2)\ (3.7\ ppm)}{20} + \frac{Vinyl\ signal\ (=CH)\ (5.6-6.6\ ppm)}{8}} * 100$$

Equation ES4: % Global monomer conversion in HPAM-MM

$$\% \, Global\ monomer\ convertion = \frac{Polyimeric\ signal\ (-CH)\ (2.1-2.5\ ppm) + \frac{PEGMA^{500}\ signal\ (-CH_2)\ (3.7\ ppm)}{20}}{Polyimeric\ signal\ (-CH)\ (2.1-2.5\ ppm) + \frac{PEGMA^{500}\ signal\ (-CH_2)\ (3.7\ ppm)}{20} + \frac{Vinyl\ signal\ (=CH)\ (5.6-6.6\ ppm)}{8}} * 100$$

Table S2. Assignation of ^{1}H NMR of copolymer HPAM and HPAM-MM synthetized after purification.

Signal (ppm)	Multiplicity	Assignation
≈ 1.6 – 2.0	Multiplet	-C**H**$_2$ (Polymer signal)
≈ 2.1 – 2.5	Multiplet	-C**H** (Polymer signal)
≈ 3.5 – 3.7	Multiplet	-C**H**$_2$ (PEGMA^{500} signal)

Equation ES5: % PEGMA[500] in the copolymer structure

$$\% \, PEGMA^{500} \, in \, the \, copolymer \, structure = \frac{\frac{PEGMA^{500} \, signal \, (-CH_2) \, (3.7 \, ppm)}{20}}{Polyimeric \, signal \, (-CH) \, (2.1-2.5 \, ppm) + \frac{PEGMA^{500} \, signal \, (-CH_2) \, (3.7 \, ppm)}{20}} * 100$$

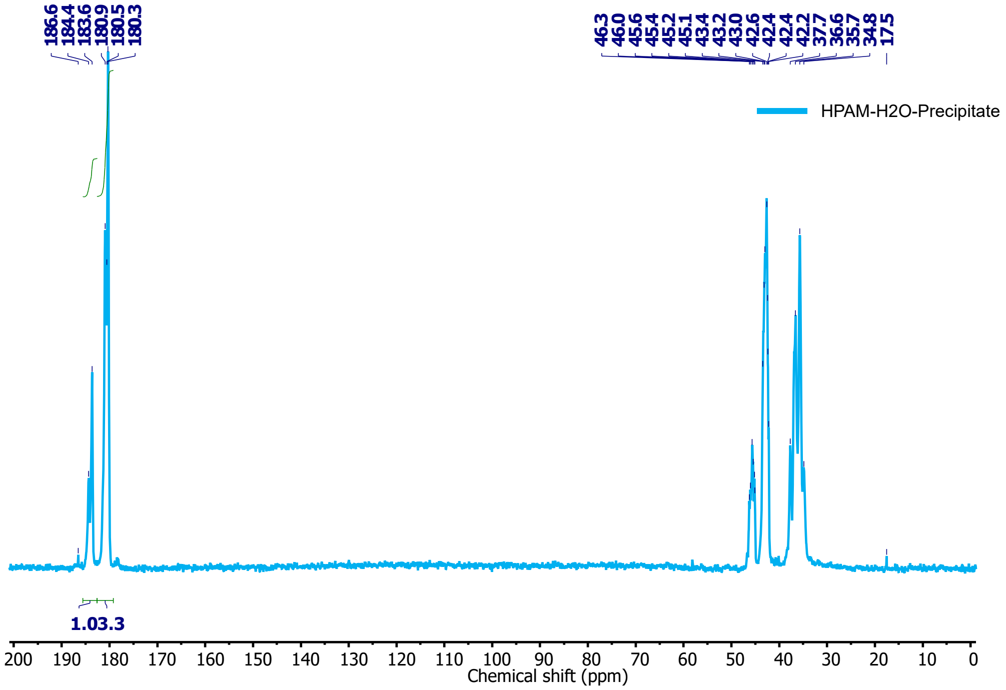

Figure S9. ^{13}C NMR of copolymer HPAM-H_2O-Precipitate.

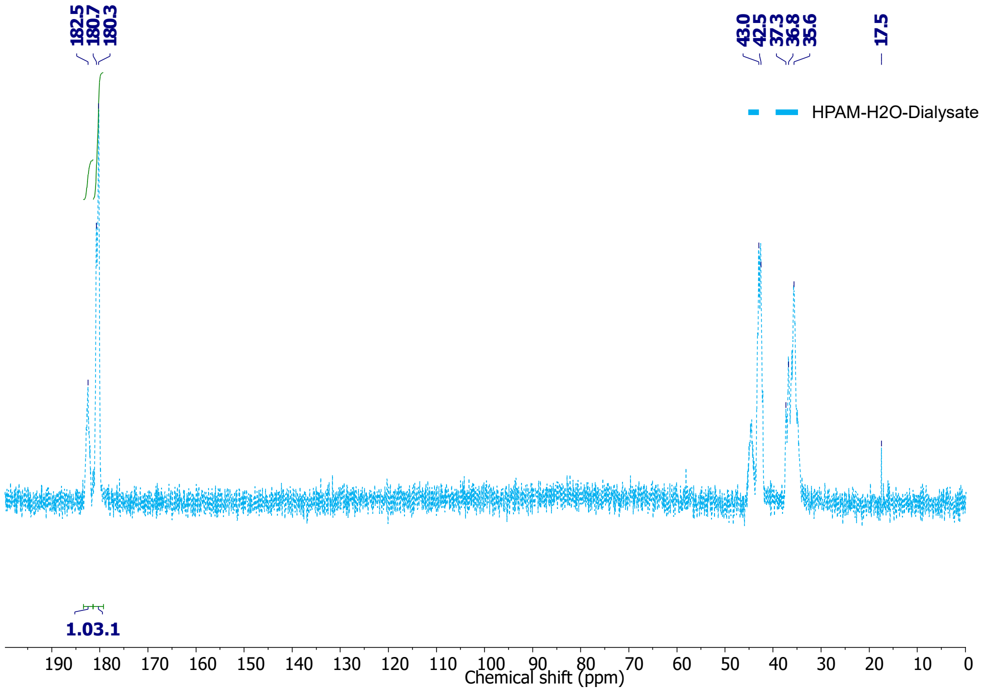

Figure S10. ^{13}C NMR of copolymer HPAM-H_2O-Dialyzate.

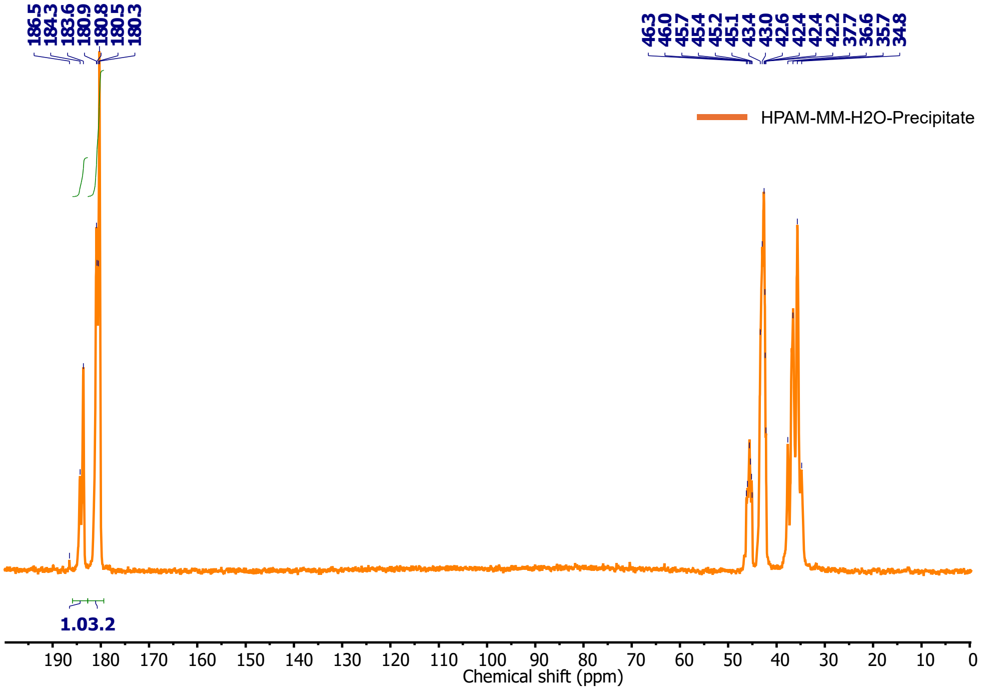

Figure S11. ^{13}C NMR of copolymer HPAM-MM-H_2O-Precipitate.

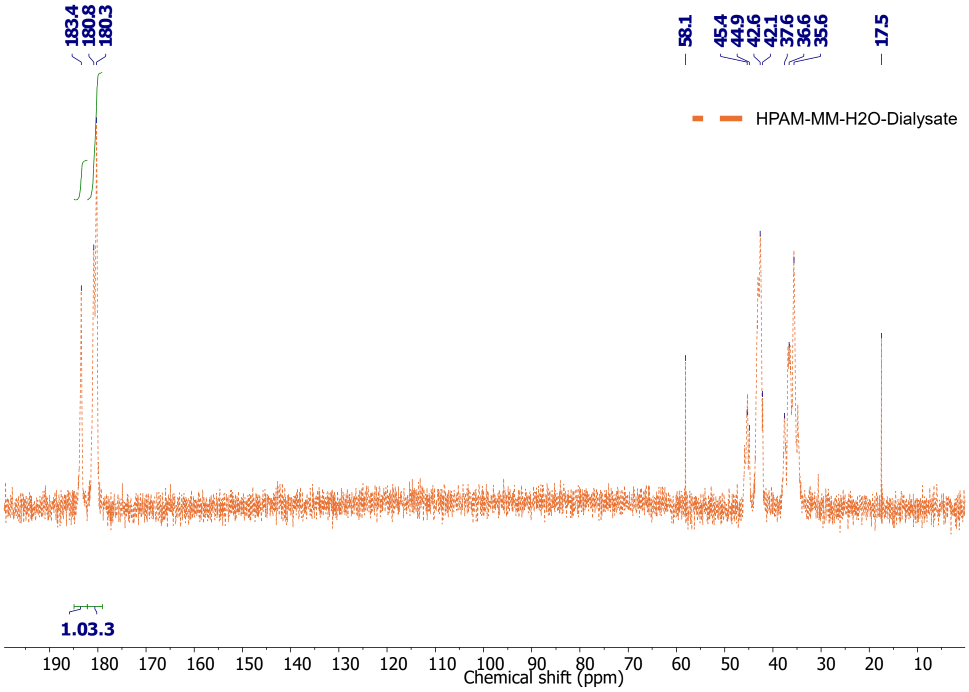

Figure S12. ^{13}C NMR of copolymer HPAM-MM-H_2O-Dialyzate.

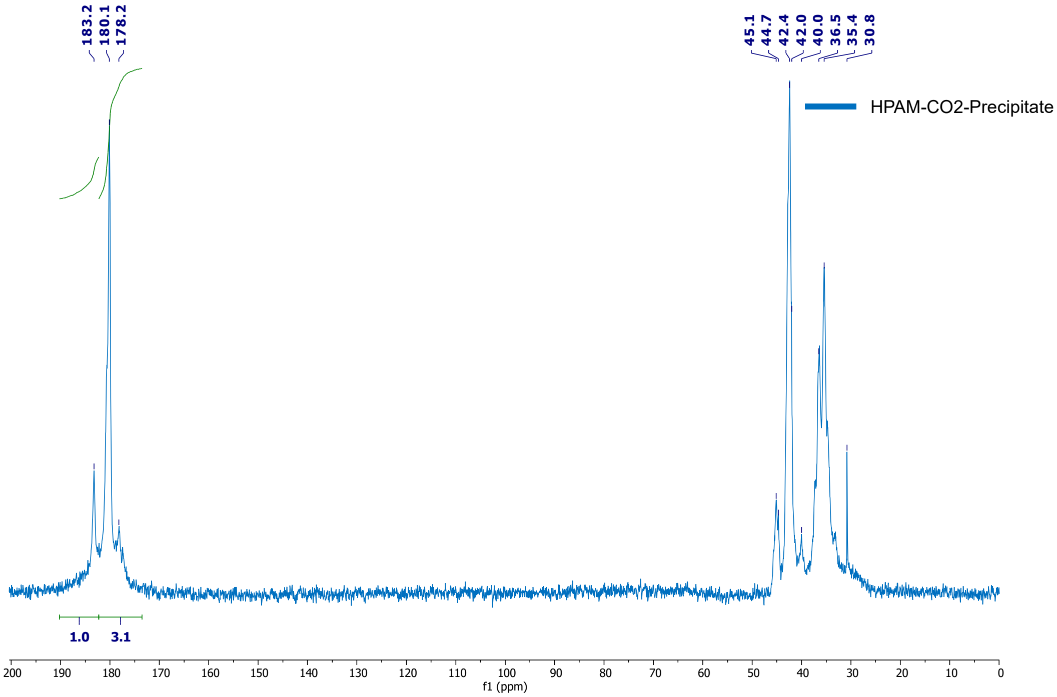

Figure S13. ^{13}C NMR of copolymer HPAM-CO_2-Precipitate.

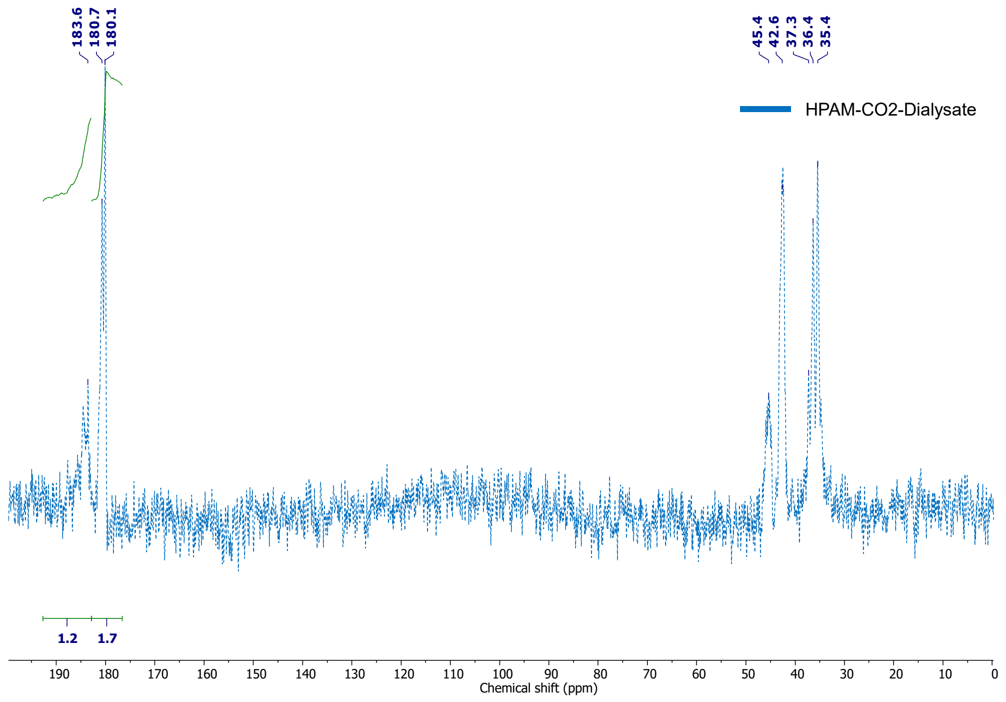

Figure S14. ^{13}C NMR of copolymer HPAM-CO_2-Dialyzate.

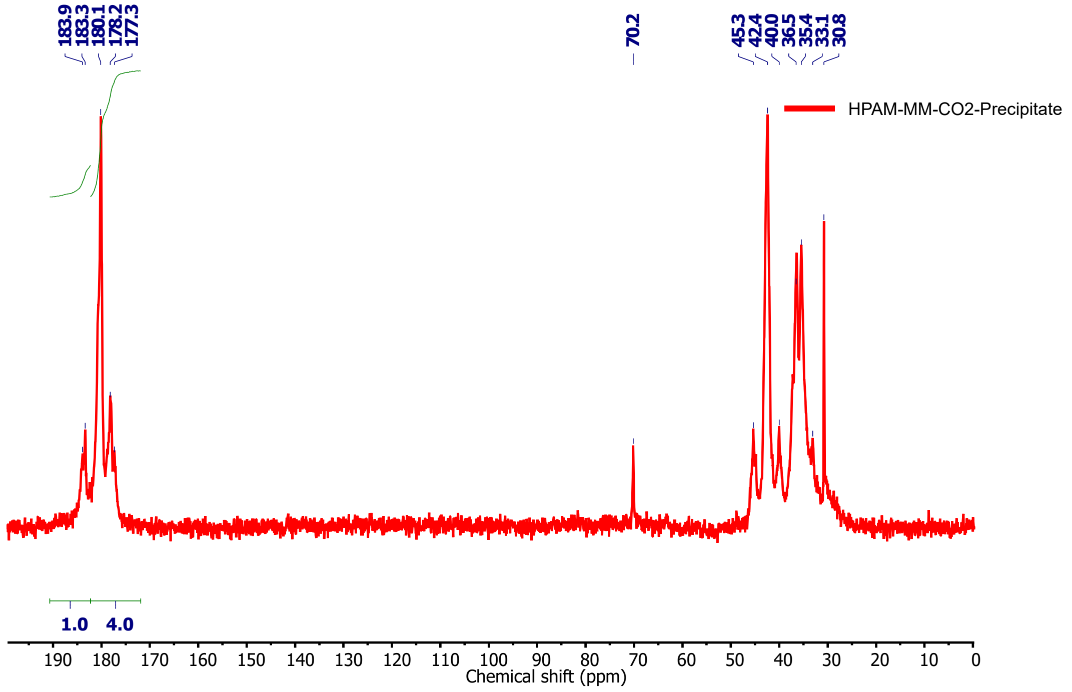

Figure S15. ^{13}C NMR of copolymer HPAM-MM-CO_2-Precipitate.

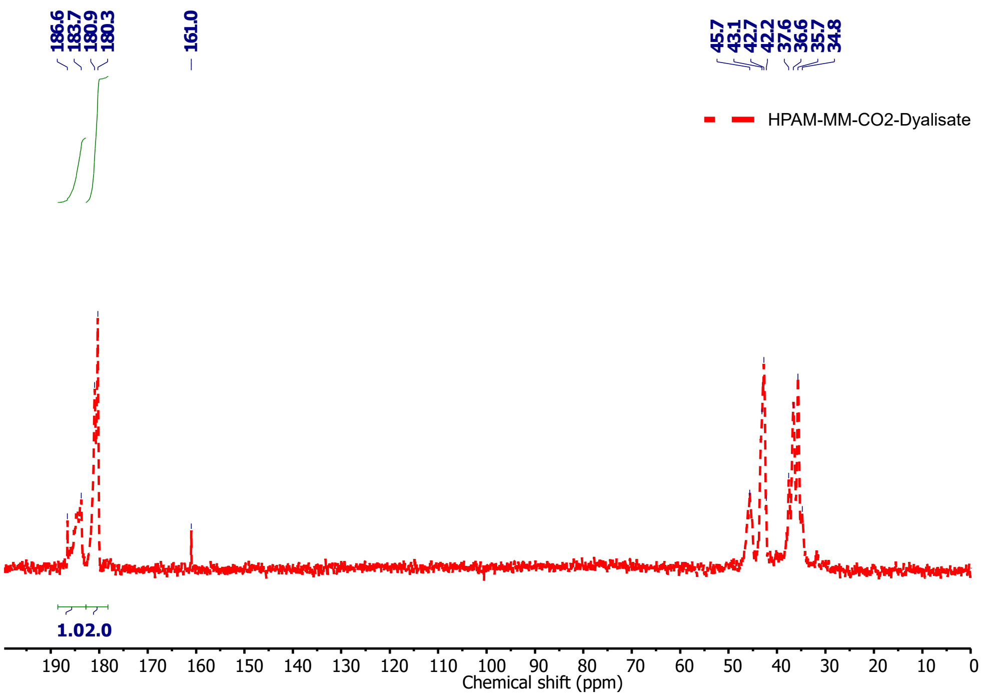

Figure S16. ^{13}C NMR of copolymer HPAM-MM-CO_2-Dialyzate.

Equation ES6: % Acrylic acid in the copolymer structure

$$\%\ Acrilic\ acid\ in\ the\ copolymer\ structure = \frac{Carbonyl\ signal\ acrylic\ acid\ (183\ ppm)}{Carbonyl\ signal\ acrylic\ acid\ (183\ ppm) + Carbonyl\ signal\ acrylamide\ (180\ ppm)} * 100$$

FTIR spectroscopy of the synthesized copolymers after purification

Applied methodology: FTIR Analysis: Peak Deconvolution

The following procedure was used for the deconvolution of the FTIR spectra

1. Absorbance conversion
2. Linear baseline correction
3. Isolation of the region of interest (e.g. 3800-2600 cm-1) (**Figure S17**, first row)
4. Calculation of the second derivative with the Savitsky-Golay smoothing filter with 21 points and a polynomial order of 3
5. Inversion of the second derivative and peak finding to get the position of each peak (**Figure S17**, second row)
6. Fourier Self-Deconvolution (FSD) using a bandwidth equal to the maximum FWHH of the peaks in the region. (a simple peak fitting without any restriction was done previously to define the FWHH) **Figure S17**, third row)
7. Comparison of the detected peaks by the two different mentioned approaches, and selection of the most realistic ones based on the proposed molecular structure and interactions in the polymer.
8. Gauss/Lorentzian peak fitting by using the positions of the most probable peaks defined previously (**Figure S17**, fourth row) with a restriction of the center of the peak of ± 10 cm^{-1}.

   All fitting procedures were carried out using OMNIC 8.2. Software (Thermo Fisher Scientific Inc.) stopped when a standard error of less than 2% was obtained.

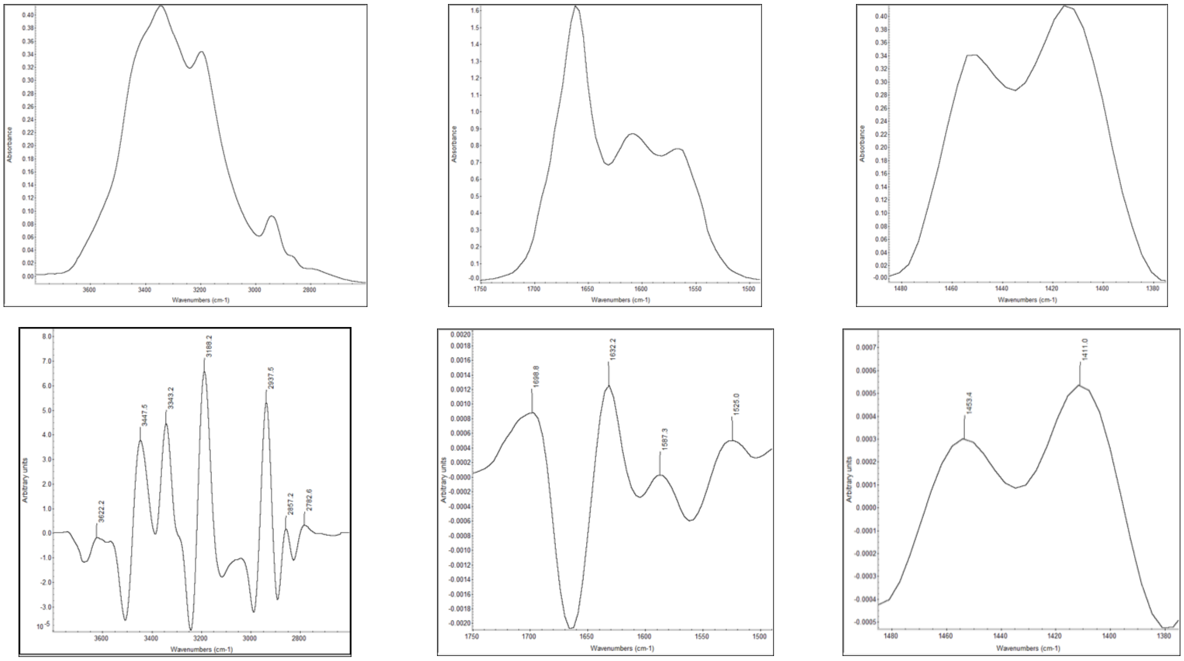

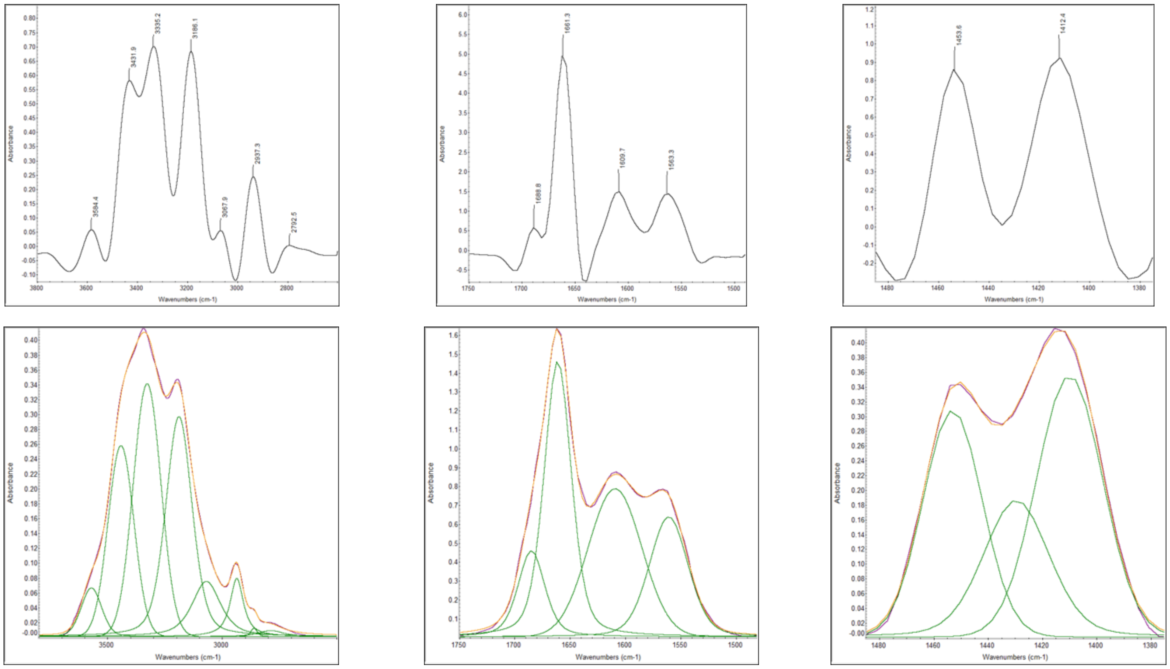

Figure S17. Deconvolution procedure for HPAM-H2O in the three relevant regions: N-H stretching region betweeen 3800 and 2600 cm^{-1} **(first column)**, carbonyl region between 1800 and 1500 cm^{-1} **(second column)**, and C-N stretching region 1375 and 1485 cm^{-1} **(third column)**. Original spectrum (black), Fitted spectrum (orange), deconvoluted peaks (green).

HPAM-H2O

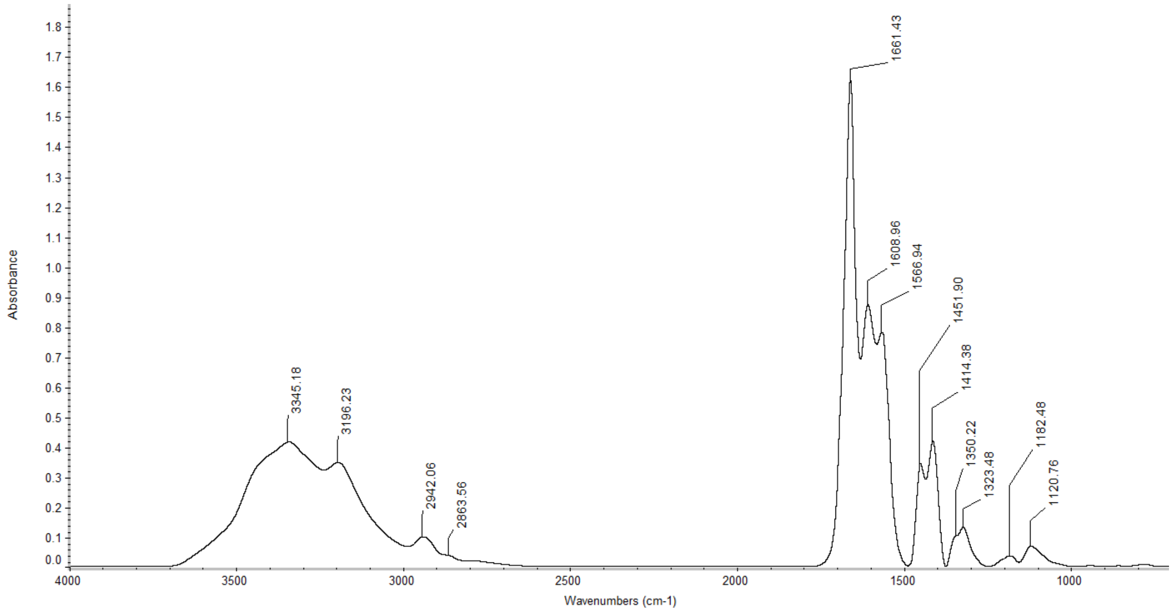

Figure S18. FTIR Absorbance spectrum of HPAM-H2O

Table S3. Peak deconvolution of HPAM-H2O in the region 3800 cm^{-1} – 2600 cm^{-1}

Peak	Maximum	FWHH	Height	Area	Assignation
1	2788.1	128.0	0.008	1.55	C-H stretching (CH)
2	2862.2	32.7	0.012	0.50	C-H stretching (CH_2)
3	2937.3	69.4	0.079	8.21	NH_2 sym. stretching (H-bonded N-H--O-C=O)
4	3070.0	147.0	0.074	15.09	NH_2 sym. stretching (H-bonded N-H--O=C-)
5	3189.8	135.1	0.297	49.03	NH_2 asym. stretching (H-bonded N-H--O-C=O)
6	3328.7	142.5	0.341	51.71	NH_2 asym. stretching (H-bonded N-H--O=C-)
7	3442.9	132.5	0.258	36.32	N-H stretching (Free)

Table S4. Peak deconvolution of HPAM-H2O in the region 1750 cm^{-1} – 1480 cm^{-1}

Peak	Maximum	FWHH	Height	Area	Assignation
1	1561.0	41.5	0.637	30.97	COO^- asymmetric stretching
2	1609.7	57.1	0.789	47.95	NH_2 bending
3	1661.3	28.9	1.455	51.61	C=O stretching (H-bonded Amide)
4	1685.1	28.9	0.466	16.86	C=O stretching (H-bonded COOH)
5	1721.4	7.7	0.000	0.00	C=O stretching (Free)**

** All initial conditions yielded a 0-height peak for signals between 1715 and 1735 corresponding to non-hydrogen bonded carbonyl (C=O stretching (Free)).

Table S5. Peak deconvolution of HPAM-H2O in the region 1485 cm^{-1} – 1375 cm^{-1}

Peak	Maximum	FWHH	Height	Area	Assignation
1	1413.1	33.8	0.42	15.18	C-N stretching (Amide III)
2	1451.4	28.6	0.33	10.10	C-H bending (CH_2)

As reported in [1] the asymmetric stretching band of the acrylate/acrylic acid group in poly(acrylic acid-*co*-acrylamide) is hidden and cannot be detected by any of the proposed deconvolution methods, by forcing a peak in the corresponding region as the authors proposed, the following results are obtained (**Figure S14**).

Table S6. Peak deconvolution of HPAM-H2O in the region 1485 cm^{-1} – 1375 cm^{-1} forcing a hidden peak in the region 1405-1410.

Peak	Maximum	FWHH	Height	Area	Assignation
1	1410.0	28.7	0.355	10.83	Symmetric COO^- stretching
2	1429.7	28.6	0.186	6.18	C-N stretching (Amide III)
3	1453.2	25.6	0.308	8.41	C-H bending (CH_2)

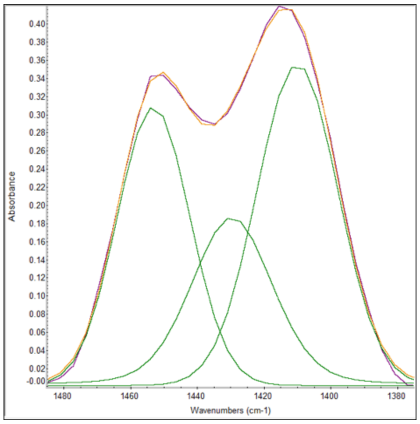

Figure S19. Peak deconvolution of HPAM-H2O in the region 1485 cm-1 – 1375 cm-1 forcing a hidden peak in the region 1405-1410

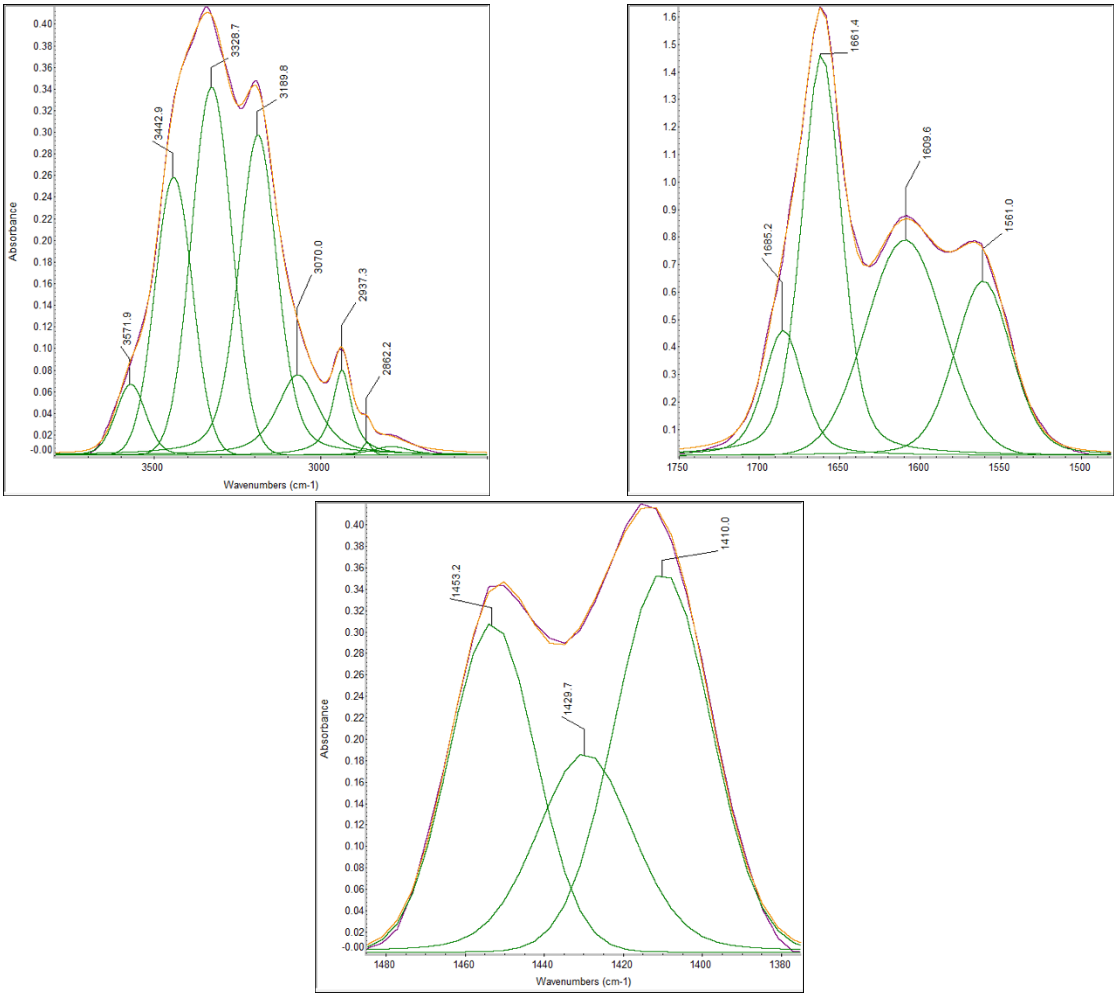

Figure S20. Peak deconvolution of HPAM-H2O in the three regions N-H stretching region between 3800 and 2600 cm-1 (**top left**), carbonyl region between 1800 and 1500 cm^{-1} (**top right**), and the C-N stretching region 1375 and 1485 cm-1 (**bottom**). Original spectrum (black), Fitted spectrum (orange), deconvoluted peaks (green).

The spectra of all copolymers were analyzed as described obtaining the following results.

HPAM-MM-H2O

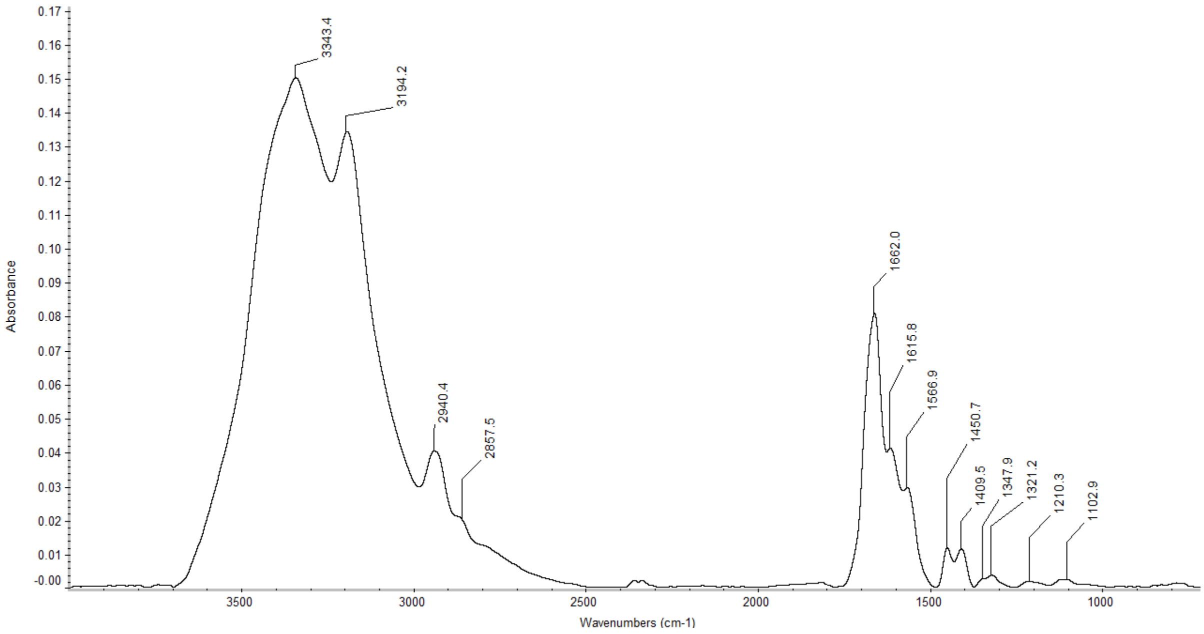

Figure S21. FTIR Absorbance spectrum of HPAM-MM-H2O

Table S7. Peak deconvolution of HPAM-MM-H2O in the region 3800 cm^{-1} – 2600 cm^{-1}

Peak	Maximum	FWHH	Height	Area	Assignation
1	2861.5	55.8	0.015	1.21	C-H stretching (CH)
2	2937.8	76.5	0.081	8.97	C-H stretching (CH_2)
3	3075.6	149.9	0.079	14.97	NH_2 sym. stretching (H-bonded N-H--O-C=O)
4	3191.4	127.3	0.216	32.95	NH_2 sym. stretching (H-bonded N-H--O=C-)
5	3338.6	164.5	0.274	48.02	NH_2 asym. stretching (H-bonded N-H--O-C=O)
6	3453.0	120.9	0.155	21.74	NH_2 asym. stretching (H-bonded N-H--O=C-)
7	3565.1	102.7	0.043	4.86	N-H stretching (Free)

Table S8. Peak deconvolution of HPAM-MM-H2O in the region 1750 cm^{-1} – 1480 cm^{-1}

Peak	Maximum	FWHH	Height	Area	Assignation
1	1554.6	41.0	0.112	5.52	COO^- asymmetric stretching
2	1605.5	61.4	0.130	8.48	NH_2 bending
3	1656.6	33.6	0.197	7.05	C=O stretching (H-bonded Amide)
4	1683.8	31.3	0.101	3.77	C=O stretching (H-bonded COOH)
5	1723.5	26.3	0.000	0.00	C=O stretching (Free)**

** All initial conditions yielded a 0-height peak for signals between 1715 and 1735 corresponding to non-hydrogen bonded carbonyl (C=O stretching (Free)).

Table S9. Peak deconvolution of HPAM-MM-H2O in the region 1485 cm^{-1} – 1375 cm^{-1} forcing a hidden peak in the region 1405-1410.

Peak	Maximum	FWHH	Height	Area	Assignation
1	1410.0	28.7	0.355	10.83	Symmetric COO^- stretching
2	1429.7	28.6	0.186	6.18	C-N stretching (Amide III)
3	1453.2	25.6	0.308	8.41	C-H bending (CH_2)

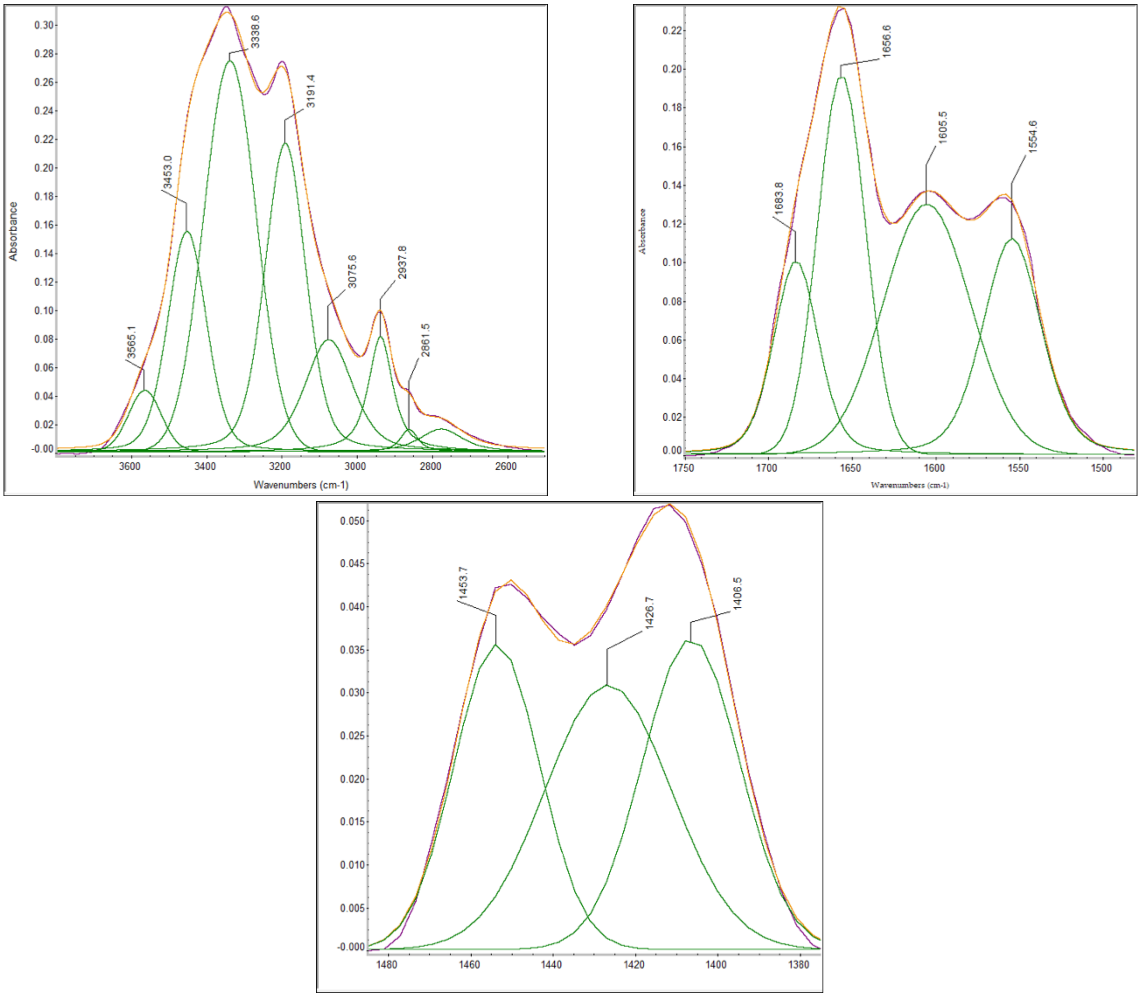

Figure S22. Peak deconvolution of HPAM-MM-H2O in the three regions N-H stretching region between 3800 and 2600 cm-1 (**top left**), carbonyl region between 1800 and 1500 cm-1 (**top right**), and the C-N stretching region 1375 and 1485 cm-1 (**bottom**). Original spectrum (black), Fitted spectrum (orange), deconvoluted peaks (green).

HPAM-CO2

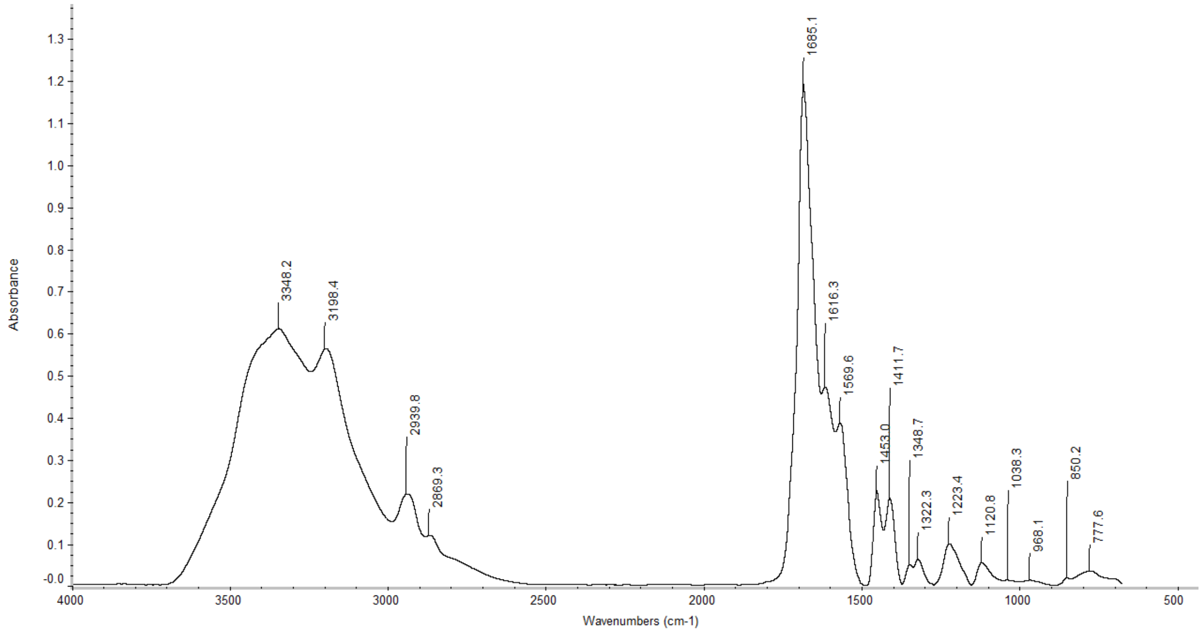

Figure S23. FTIR Absorbance spectrum of HPAM-CO2

Table S10. Peak deconvolution of HPAM-CO2 in the region 3800 cm^{-1} – 2600 cm^{-1}

Peak	Maximum	FWHH	Height	Area	Assignation
1	2854.8	30.7	0.024	1.15	C-H stretching (CH)
2	2938.1	118.5	0.182	39.55	C-H stretching (CH_2)
3	3063.4	80.4	0.077	6.58	NH_2 sym. stretching (H-bonded N-H--O-C=O)
4	3190.9	158.8	0.495	103.91	NH_2 sym. stretching (H-bonded N-H--O=C-)
5	3316.1	126.9	0.315	50.43	NH_2 asym. stretching (H-bonded N-H--O-C=O)
6	3422.0	166.3	0.461	81.60	NH_2 asym. stretching (H-bonded N-H--O=C-)
7	3570.0	104.8	0.089	9.89	N-H stretching (Free)

Table S11. Peak deconvolution of HPAM-CO2 in the region 1750 cm^{-1} – 1480 cm^{-1}

Peak	Maximum	FWHH	Height	Area	Assignation
1	1566.4	49.1	0.360	18.83	COO^- asymmetric stretching
2	1615.5	45.5	0.429	20.76	NH_2 bending
3	1663.3	41.5	0.769	34.01	C=O stretching (H-bonded Amide)
4	1688.1	27.6	0.773	22.69	C=O stretching (H-bonded COOH)
5	1713.0	41.3	0.335	14.73	C=O stretching (Free)

Table S12. Peak deconvolution of HPAM-CO2 in the region 1485 cm^{-1} – 1375 cm^{-1} forcing a hidden peak in the region 1405-1410.

Peak	Maximum	FWHH	Height	Area	Assignation
1	1407.8	28.2	0.173	5.20	COO^- symmetric stretching
2	1430.0	33.2	0.088	3.10	C-N stretching (Amide III)
3	1453.8	23.2	0.196	4.83	CH_2 bending

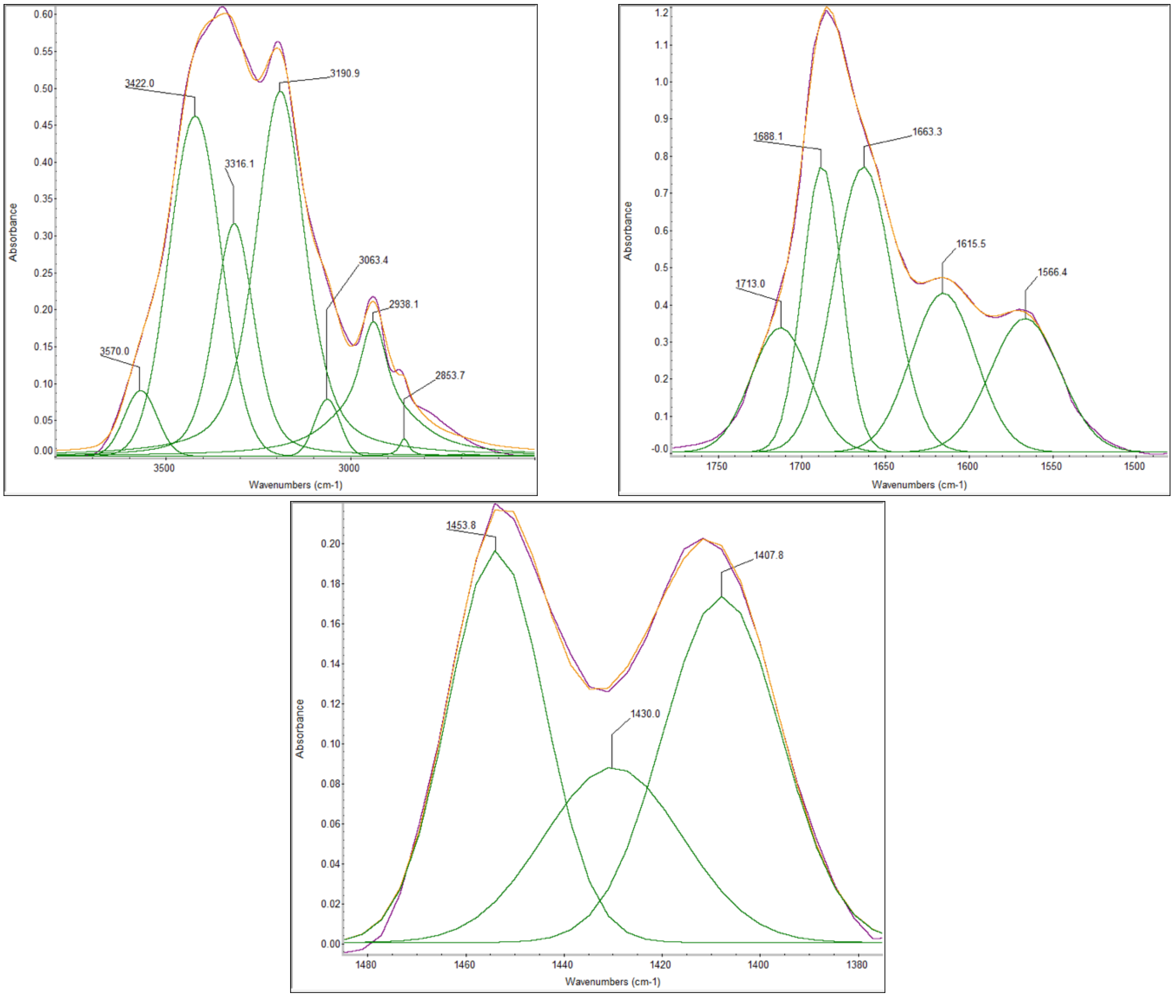

Figure S24. Peak based deconvolution of HPAM-CO2 in the three regions: N-H stretching region betweeen 3800 and 2600 cm^{-1} (**top left**), carbonyl region between 1800 and 1500 cm^{-1} (**top right**), and the C-N stretching region 1375 and 1485 cm^{-1} (**bottom**). Original spectrum (black), Fitted spectrum (orange), deconvoluted peaks (green).

HPAM-MM-CO2

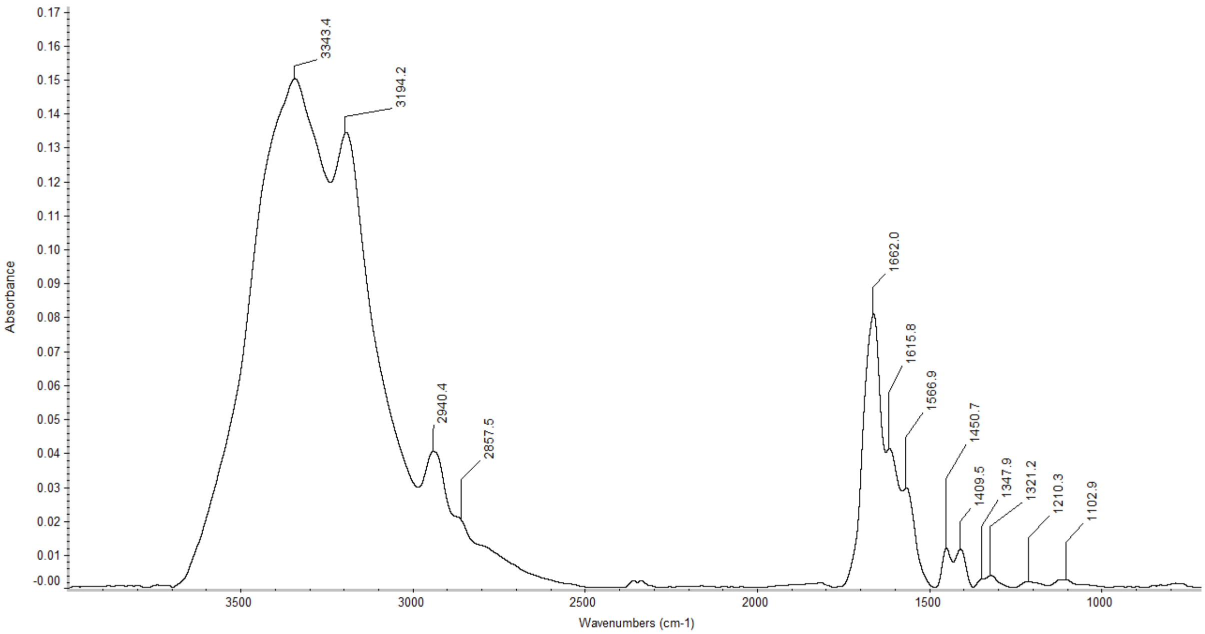

Figure S25. FTIR Absorbance spectrum of HPAM-MM-CO2

Table S13. Peak deconvolution of HPAM-MM-CO2 in the region 3800 cm^{-1} – 2600 cm^{-1}

Peak	Maximum	FWHH	Height	Area	Assignation
1	2857.5	63.7	0.007	0.62	C-H stretching (CH)
2	2937.3	80.8	0.031	3.52	C-H stretching (CH_2)
3	3068.0	127.8	0.033	5.52	NH_2 sym. stretching (H-bonded N-H--O-C=O)
4	3185.4	127.4	0.112	18.35	NH_2 sym. stretching (H-bonded N-H--O=C-)
5	3325.0	153.3	0.117	19.07	NH_2 asym. stretching (H-bonded N-H--O-C=O)
6	3439.0	144.1	0.087	14.93	NH_2 asym. stretching (H-bonded N-H--O=C-)
7	3562.1	96.1	0.020	2.28	N-H stretching (Free)

Table S14. Peak deconvolution of HPAM-MM-CO2 in the region 1750 cm^{-1} – 1480 cm^{-1}

Peak	Maximum	FWHH	Height	Area	Assignation
1	1562.5	48.0	0.025	1.38	COO^- asymmetric stretching
2	1613.7	49.2	0.037	2.20	NH_2 bending
3	1656.8	34.2	0.060	2.43	C=O stretching (H-bonded Amide)
4	1682.9	37.7	0.050	1.99	C=O stretching (H-bonded COOH)
5	1721.9	21.4	0.005	0.13	C=O stretching (Free)

Table S15. Peak deconvolution of HPAM-MM-CO2 in the region 1485 cm^{-1} – 1375 cm^{-1} forcing a hidden peak in the region 1405-1410.

Peak	Maximum	FWHH	Height	Area	Assignation
1	1405.2	27.9	0.009	0.27	Symmetric COO^- stretching
2	1426.5	35.3	0.006	0.22	C-N stretching (Amide III)
3	1452.6	23.8	0.011	0.26	C-H bending (CH_2)

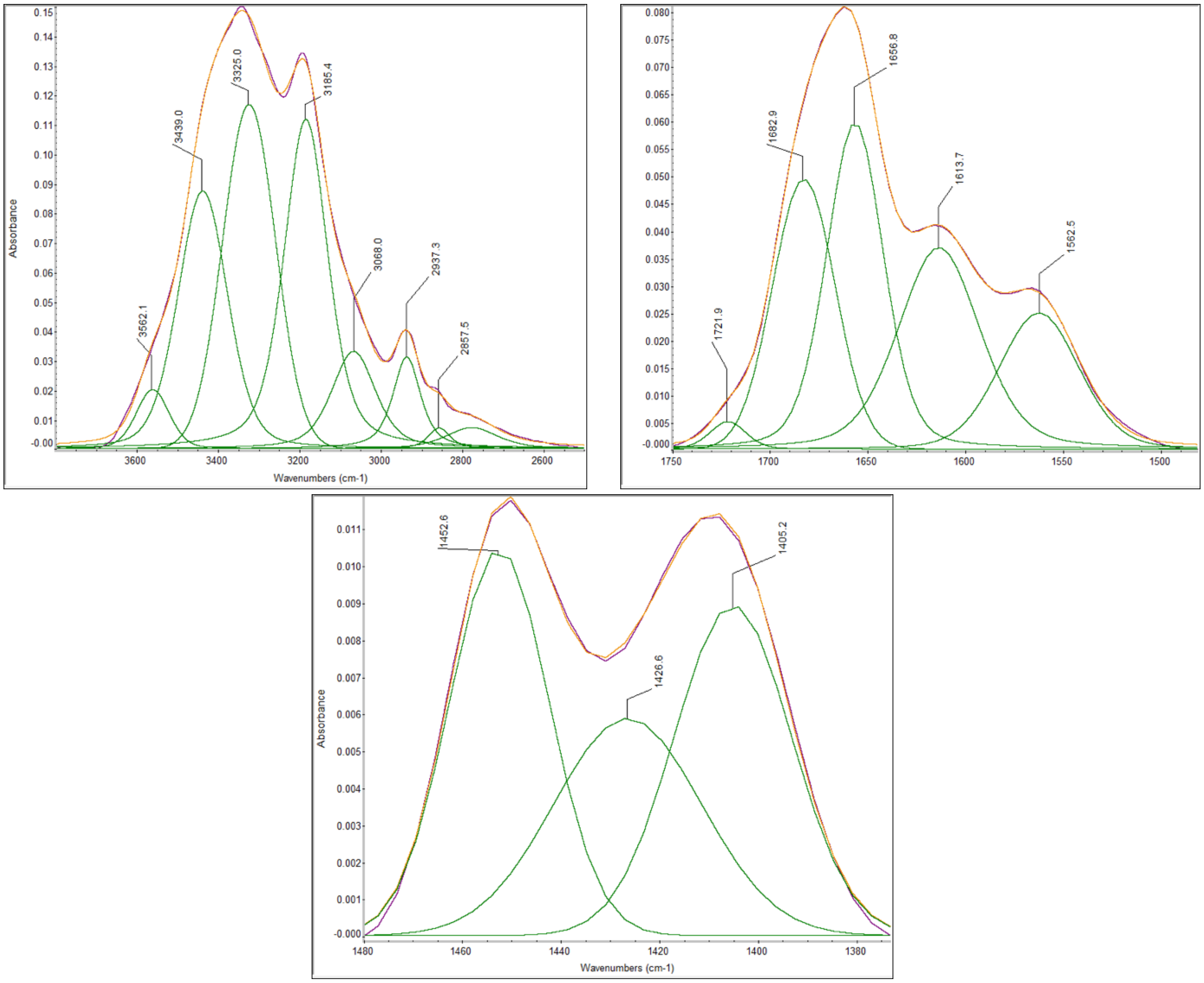

Figure S26. Peak deconvolution of HPAM-MM-CO2 in the regions: N-H stretching region betweeen 3800 and 2600 cm^{-1} (**top left**), carbonyl region between 1800 and 1500 cm^{-1} (**top right**), and the C-N stretching region 1375 and 1485 cm^{-1} (**bottom**). Original spectrum (black), Fitted spectrum (orange), deconvoluted peaks (green).

Size exclusion chromatography, multi-angle light scattering and differential refractometer index detectors of the synthesized copolymers after purification

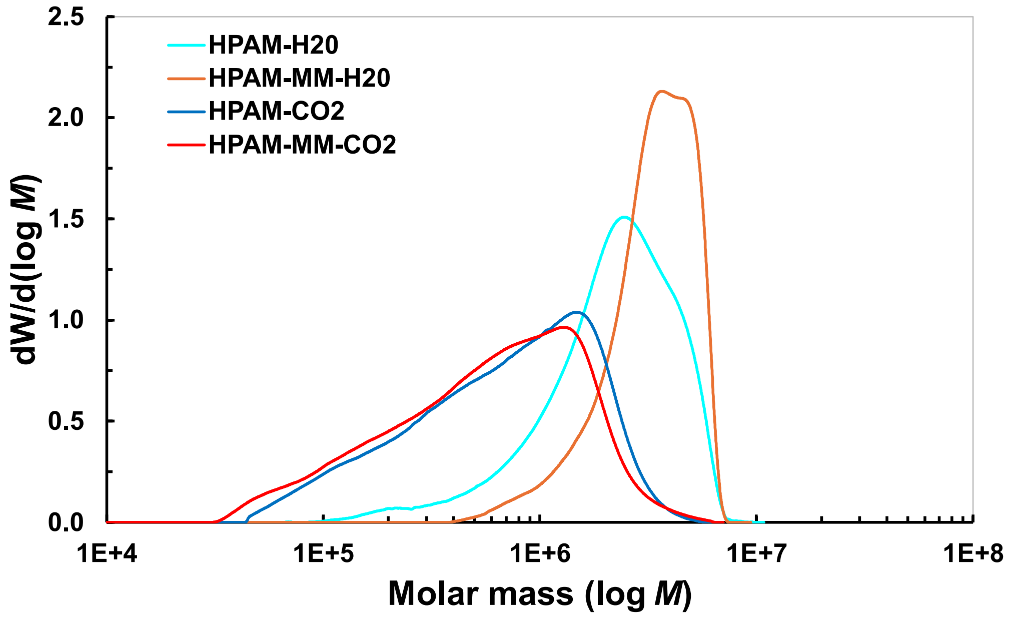

Figure S27. SEC-MALS-dRI: Differential molar mass distribution of synthesized copolymers studied by precipitation purification method.

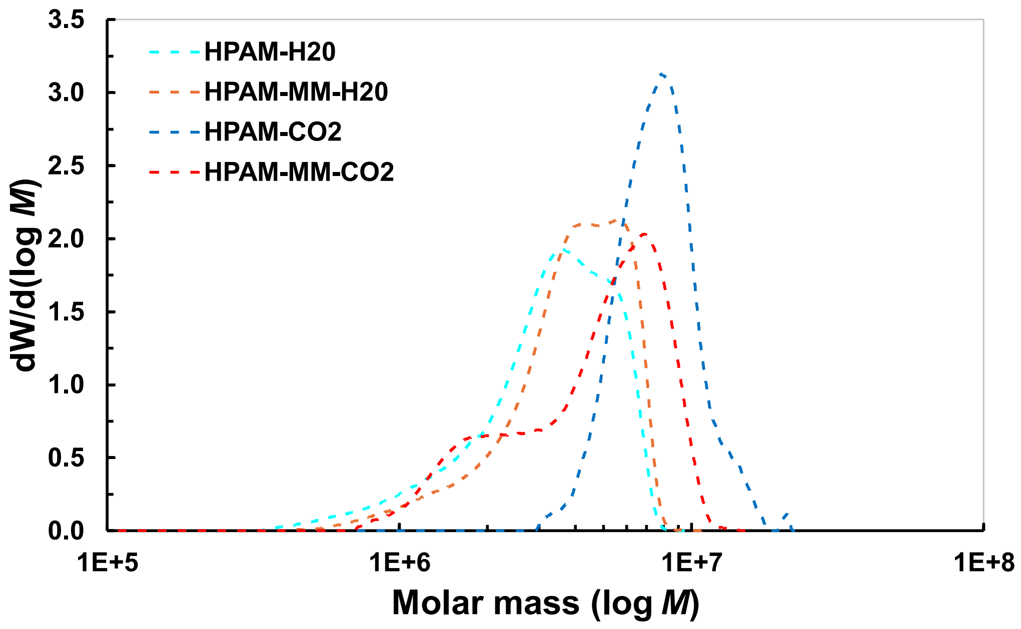

Figure S28. SEC-MALS-dRI: Differential molar mass distribution of synthesized copolymers studied by dialysate purification method.

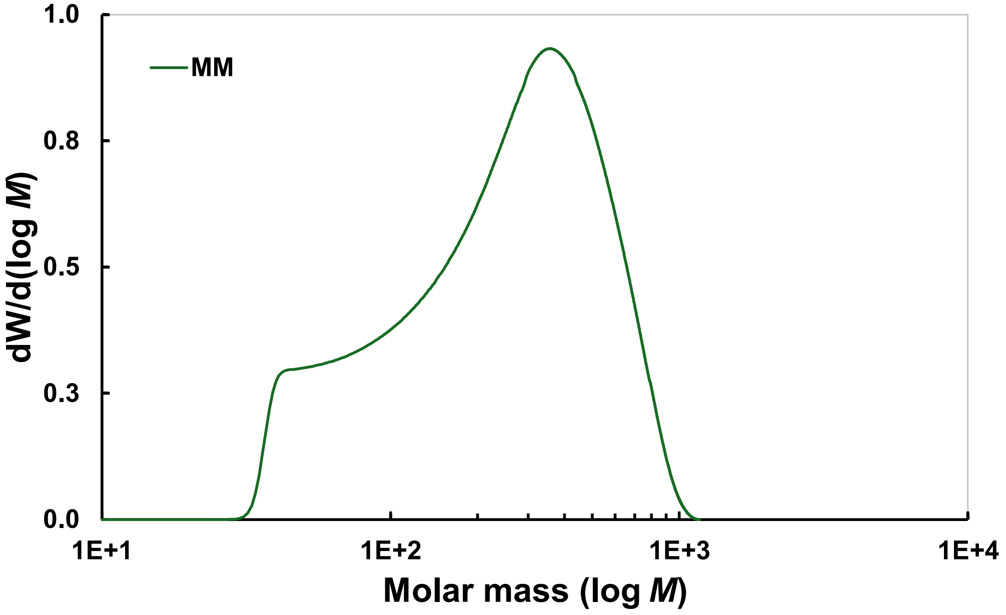

Figure S29. SEC-MALS-dRI: Differential molar mass distribution of MM.

Dynamic light scattering of the synthesized copolymers after purification

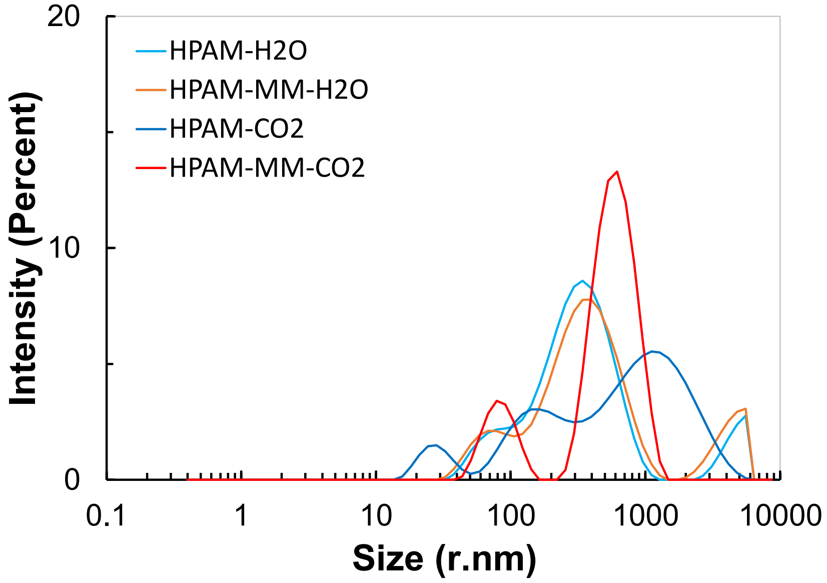

Figure S30. DLS intensity-based hydrodynamic radius analysis of the synthesized copolymers in ultra-pure water filters after purification for precipitation. [Copolymer]=100 mg L^{-1}. Temperature: 25 °C.

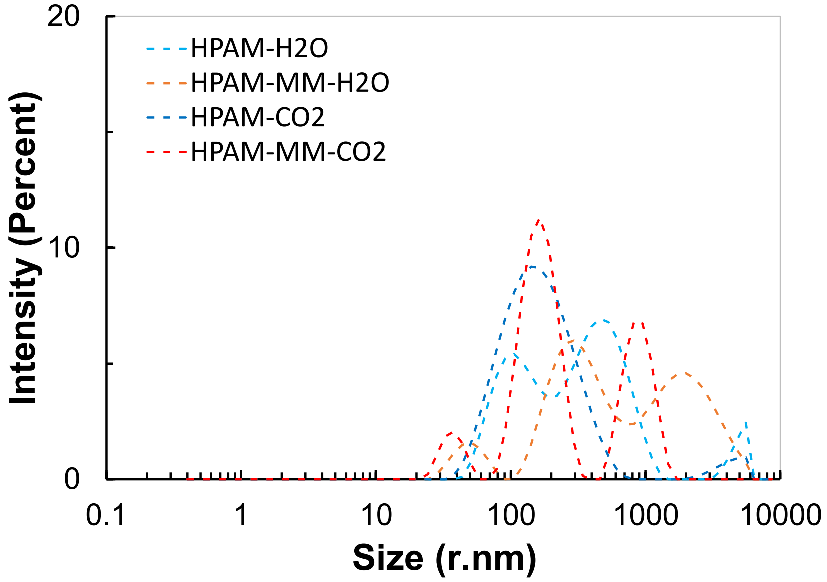

Figure S31. DLS intensity-based hydrodynamic radius analysis of the synthesized copolymers in ultra-pure water filters after purification for dialysis. [Copolymer]=100 mg L^{-1}. Temperature: 25 °C.

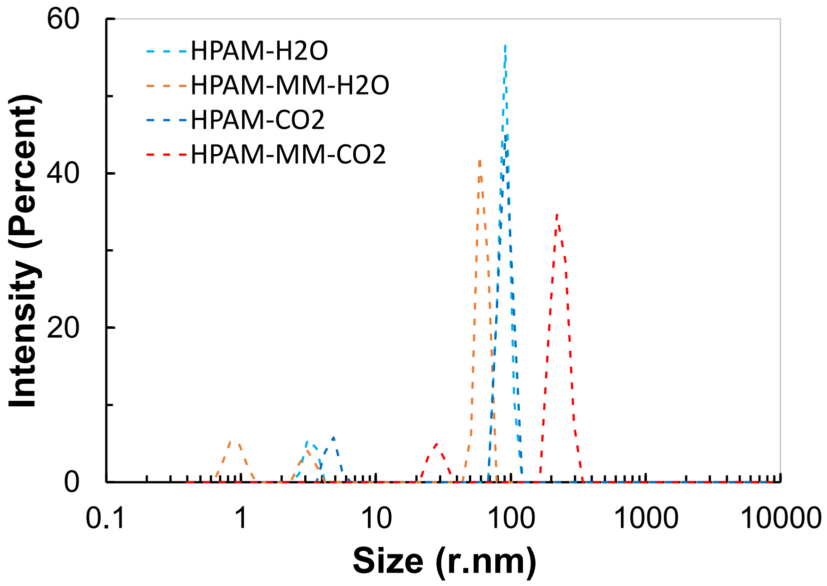

Figure S32. DLS intensity-based hydrodynamic radius analysis of the synthesized copolymers in ultra-pure water aggregate after purification for dialysate. [Copolymer]=2500 mg L^{-1}. Temperature: 25 °C.

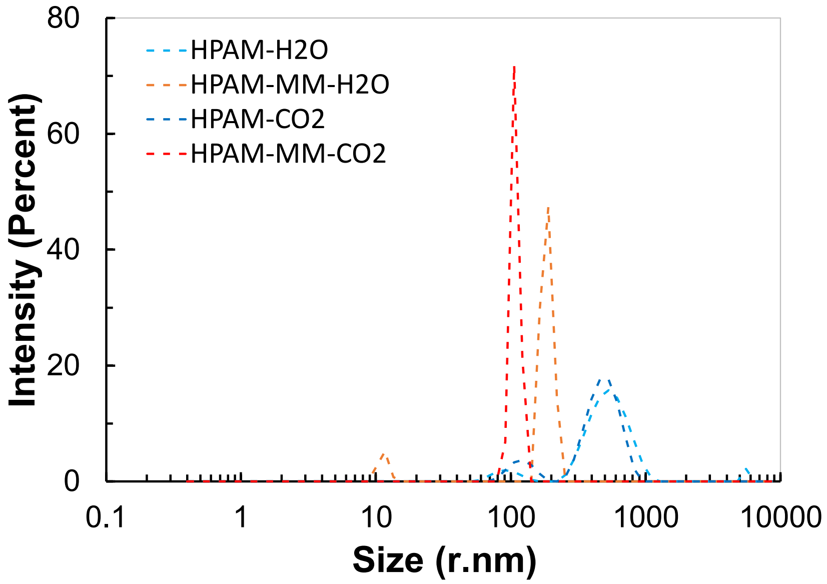

Figure S33. DLS intensity-based hydrodynamic radius analysis of the synthesized copolymers in ultra-pure water sonicate after purification for dialysate. [Copolymer]=2500 mg L^{-1}. Temperature: 25 °C.

Apparent viscosity of the synthesized copolymers in ultra-pure water

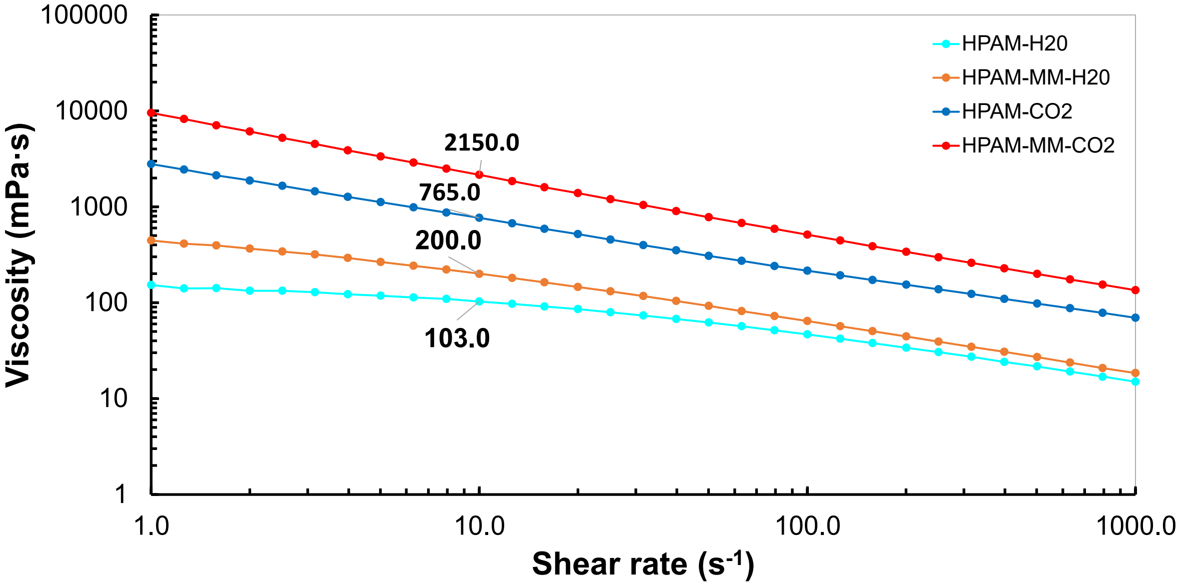

Figure S34. Apparent viscosity of synthetized copolymers in ultra-pure water after purification for dialysate. [Copolymer]=2500 mg L^{-1}, temperature: 25 °C.

DSC thermograms of the synthesized copolymers after purification

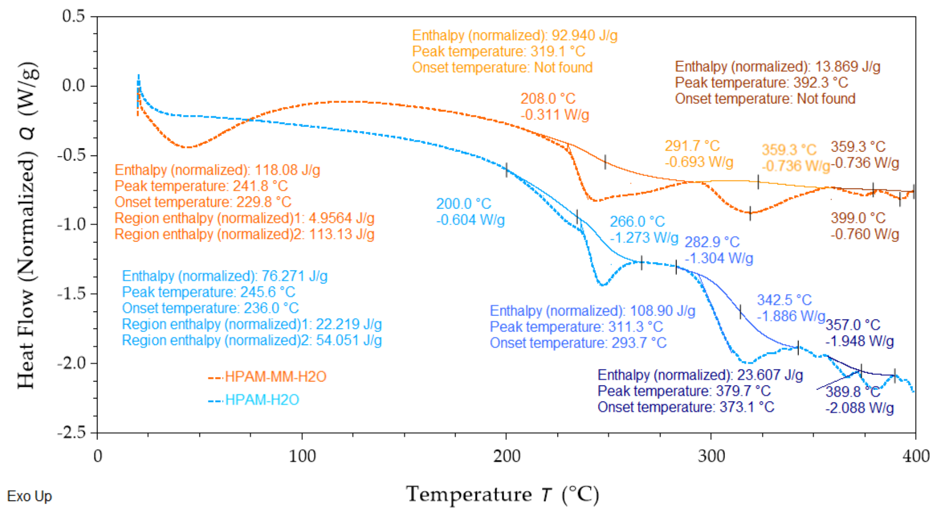

Figure S35. DSC thermograms analyzed of synthetized copolymers in aqueous solutions.

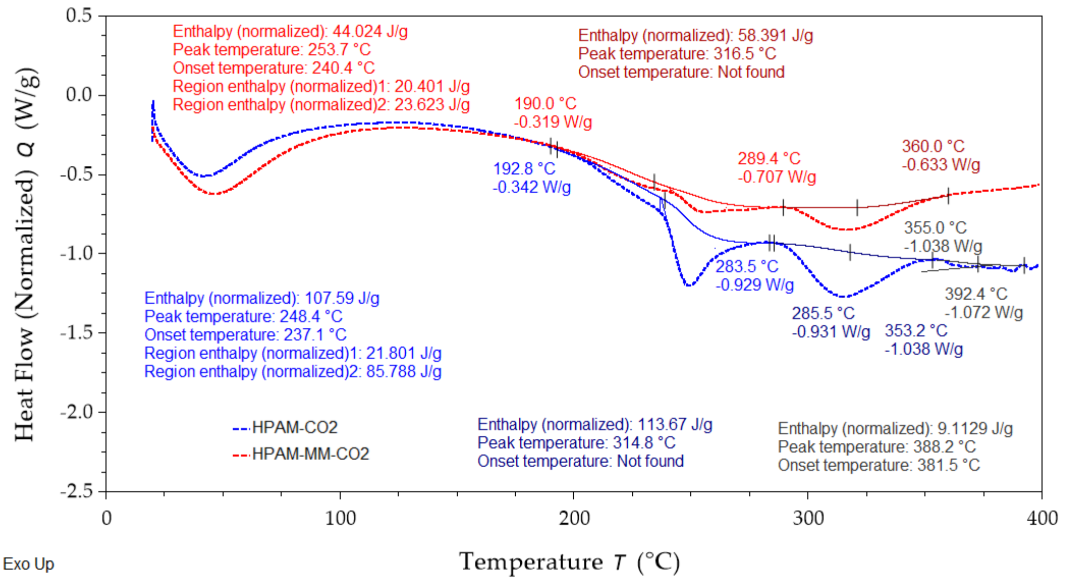

Figure S36. DSC thermograms analyzed of synthetized copolymers in pressurized CO2-ethyl acetate mixture.

References

1. Lee, Y.C.; Liew, C.W.; Buraidah, M.H.; Woo, H.J. Fourier Transform Infrared Studies of Gel Polymer Electrolyte Based on Poly(Acrylamide-Co-Acrylic Acid) – Ethylene Carbonate Incorporated with Water-Soluble Sodium Sulfide. *Opt Mater (Amst)* 2023, *140*, doi:10.1016/j.optmat.2023.113791.
